# Adverse childhood experiences and child mental health: an electronic birth cohort study

**DOI:** 10.1186/s12916-021-02045-x

**Published:** 2021-08-06

**Authors:** Emily Lowthian, Rebecca Anthony, Annette Evans, Rhian Daniel, Sara Long, Amrita Bandyopadhyay, Ann John, Mark A. Bellis, Shantini Paranjothy

**Affiliations:** 1 Population Data Science, Swansea University Medical School, Singleton Park, Swansea, SA2 8PP UK; 2 DECIPHer, 1 – 3 Museum Place, School of Social Sciences, Cardiff University, Cardiff, CF10 3BD UK; 3 Wolfson Centre for Young People’s Mental Health, Cardiff University, Cardiff, UK; 4 Division of Population Medicine, Neuadd Meirionnydd, University Hospital of Wales, Heath Park, Cardiff, CF14 4YS UK; 5 National Centre for Population Health and Wellbeing Research, Swansea University Medical School, Singleton Park, Swansea, SA2 8PP UK; 6 College of Human Sciences, Bangor University, Wrexham Technology Park, Bangor, LL13 7YP UK; 7 University of Aberdeen, Aberdeen Health Data Science Centre, Institute of Applied Health Sciences, Polwarth Building, Foresterhill, Aberdeen, AB25 2ZD UK

**Keywords:** Adverse childhood experiences, Mental health, Cohort, Wales, Survival analysis, Administrative data

## Abstract

**Background:**

Adverse childhood experiences (ACEs) are negatively associated with a range of child health outcomes. In this study, we explored associations between five individual ACEs and child mental health diagnoses or symptoms. ACEs included living with someone who had an alcohol-related problem, common mental health disorder or serious mental illness, or experienced victimisation or death of a household member.

**Methods:**

We analysed data from a population-level electronic cohort of children in Wales, UK, (*N* = 191,035) between the years of 1998 and 2012. We used Cox regression with discrete time-varying exposure variables to model time to child mental health diagnosis during the first 15 years of life. Child mental health diagnoses include five categories: (i) externalising symptoms (anti-social behaviour), (ii) internalising symptoms (stress, anxiety, depression), (iii) developmental delay (e.g. learning disability), (iv) other (e.g. eating disorder, personality disorders), and (v) any mental health diagnosis, which was created by combining externalising symptoms, internalising symptoms and other. Our analyses were adjusted for social deprivation and perinatal risk factors.

**Results:**

There were strong univariable associations between the five individual ACEs, sociodemographic and perinatal factors (e.g. gestational weight at birth) and an increased risk of child mental health diagnoses. After adjusting for sociodemographic and perinatal aspects, there was a remaining conditional increased risk of any child mental health diagnosis, associated with victimisation (conditional hazard ratio (cHR) 1.90, CI 95% 1.34–2.69), and living with an adult with a common mental health diagnosis (cHR 1.63, CI 95% 1.52–1.75). Coefficients of product terms between ACEs and deprivation were not statistically significant.

**Conclusion:**

The increased risk of child mental health diagnosis associated with victimisation, or exposure to common mental health diagnoses, and alcohol problems in the household supports the need for policy measures and intervention strategies for children and their families.

**Supplementary Information:**

The online version contains supplementary material available at 10.1186/s12916-021-02045-x.

## Background

It is well established that adverse childhood experiences (ACEs), such as abuse, neglect and household dysfunction, are negatively associated with numerous physical, social, emotional and behavioural problems in adulthood [1–3]. Up to two thirds of the population experience at least one ACE before the age of eighteen, and one quarter experience four or more [4]. Since the initial suggestion that the association between ACEs and poor outcomes was due to maladaptive coping mechanisms (e.g. substance abuse), we have seen the emergence and debate of additional mechanisms such as epigenetic and neurobiological processes that affect the development of the brain and endocrine systems [5, 6].

Childhood mental health diagnoses are relatively common, with prevalence rates being around 10% of children between the ages of 5 and 16 [7]. Understanding the relationship between exposure to ACEs and children’s behavioural and psychological outcomes is important as psychological disorders in childhood or adolescence are strong predictors of psychiatric disorders in adulthood [8]. A systematic review of empirical research on the association between ACEs and child developmental wellbeing [9] reported an association between cumulative ACEs (a count of exposures) and child behavioural problems; however, it was limited by the small number of studies, most of which included high-risk individuals (e.g. from populations receiving child welfare).

To date, ACEs have largely been examined cumulatively, and while useful, this has led to a limited understanding of which ACEs are the greatest risk for child mental health, and their relative contributions [10, 11]. The overarching literature suggests that victimisation tends to be a greater risk for internalising symptoms, and household dysfunction a larger risk for externalising symptoms [11–13]. Moreover, Hussong et al. (2007) documents that children of alcoholics are at risk for externalising symptoms, but less so for internalising symptoms [14, 15]. In terms of developmental delay, Ouyang et al. (2008) found an association between maltreatment, which included neglect and abuse, and attention-deficit disorders [16]. One study examined multiple ACEs measured individually and found that abuse, neglect, household mental illness or substance use was related to childhood depressive symptoms [10]. Due to the vast literature across areas of mental health focusing on cumulative or only one or two ACEs, there is a need to examine the risks that multiple individual ACEs pose for child mental health outcomes.

Moreover, mental health diagnoses are socially patterned, with higher rates observed among those with lower socioeconomic status or who live in areas with higher levels of social deprivation [17, 18]. Reiss (2013) conducted a systematic review and found that 52 of the 55 studies identified confirmed this relationship. They found that children from disadvantaged families were approximately two to three times more likely to develop mental health disorders. Some theoretical explanations include the social causation hypothesis, which suggests that stress associated with a low social position contributes to the development of mental health disorders [18]. This, paired with the knowledge that children from disadvantaged backgrounds are much more likely to experience an ACE [2], raises the question as to the role of ACEs in the relationship between social disadvantage and mental health outcomes. However, little research has addressed this, and it is considered a significant gap in the field [19, 20].

In this study, we address the gap in the evidence base in four ways. First, we assess the associations between individual ACEs, demographic and perinatal confounders on the rate of child mental health diagnoses and symptoms using univariable models. Second, in a single model, we investigate the conditional association of all demographic and perinatal confounders with the rate of child mental health outcomes. Third, we investigate the extent to which the conditional associations of the demographic and perinatal variables with child mental health outcomes change upon adding the individual ACE variables into the previous model. Lastly, we investigate the extent to which the available measure of deprivation moderates the associations found between individual ACEs and child mental health outcomes. The four steps are ultimately motivated by a desire to understand the effects of ACEs individually (as opposed to cumulatively) on child mental health (step 3), the extent to which these are confounded by sociodemographic factors (comparing steps 1 and 3) and the extent to which they are moderated by deprivation (step 4). Additionally, we are interested in the extent to which the five ACEs mediate the effect of sociodemographic factors on child mental health, which we will assess informally by comparing steps 2 and 3, rather than conducting a formal mediation analyis.

## Methods

### Data sources and study design

The Wales Electronic Cohort for Children (WECC) contains 981,404 children born between Jan 1, 1990, and Oct 7, 2012, for a child or mother resident in Wales [21]. WECC is derived from de-identified routinely available health and social data sets that have been record-linked and made available, to protect privacy, in the Secure Anonymised Information Linkage (SAIL) databank at Swansea University, UK [22, 23]. Individuals are allocated a unique Anonymised Linking Field based on encrypted National Health Service numbers provided by NHS Wales Informatics Service (NWIS), for each data set within the SAIL databank. The SAIL linkage system uses a both deterministic and probabilistic record-linkage with more than 99.9% accuracy. Deterministic record-linkage is based on NHS numbers and probabilistic record-linkage based on first name, surname, date of birth, gender and phonetic and soundex version of names [23]. Each child was assigned a residential anonymised linking field (RALFs) for each address during the study period, created by encrypting addresses within NWIS [24, 25], which enables anonymous linkage of those living in the same household. We used WECC data from Jan 5, 1998, to Oct 7, 2012, and this was defined by the availability of data on hospital admissions from the Patient Episode Database for Wales. This enabled measurement of ACE exposures using hospital admission and primary care data for each child, and household members that a child lived with. Each child also had perinatal variables using data from the National Community Child Health Database and outcome measures using General Practice (GP) data from Welsh Longitudinal General Practice data set. We included children who were living in Wales, for whom GP data were available. Children who had moved away or died were censored on the date they moved out of Wales (identified from the Wales Demographic Service) or died (identified from the Public Health mortality files) before Oct 12, 2012. Children were included if they had a valid RALF and adult household members with sufficient primary care data to enable ascertainment of exposure groups (See Figure 1 in Additional file 1 for participant flowchart). At the time of data extraction, the SAIL databank had data from over 40% of the 474 General Practices in Wales, over 1.9 million people.

### Measures

#### Adverse childhood experiences (ACEs)

As in other research [26], we defined five measures of ACEs. The first relates to childhood victimisation, defined using a set of ICD-10 codes in any position of a finished consultant episode of an inpatient hospital admission [27]; we did not use P codes as these are neonatal related, for codes see Additional file 13, Table 11. Three further ACEs relate to household dysfunction and were defined as the presence of any of the following in an adult household member: (i) serious mental illness diagnosis (e.g. bipolar, schizophrenia) [28], (ii) common mental disorder (e.g. depression, anxiety) [29] and (iii) problematic alcohol use ascertained using a set of primary care READ codes for heavy drinker, ex-heavy drinker, alcohol dependence, alcoholic liver disease, alcohol related nervous system or stomach problems, poisoning or treatment evidence [30] and/or any alcohol-related emergency hospital admission during the exposure period [31]. The fifth ACE was the date of death of a household member, given that the loss of a parent, sibling or other non-parent household member is likely to be a momentous event [32].

Household members were defined as those who were living in the same household as the child on their on their 1st, 5th or 8th birthday. For household members who were living with the child on their first birthday, we also ascertained if there was any history of a common mental disorder or serious mental illness from 1998 onwards. We created four exposure periods: birth to <1 year, 1≤5 years, 5≤8 years and 8≤12 years. The presence or absence of each of the five ACEs as defined above, in each exposure period, was ascertained from a search for the relevant READ or ICD-10 codes for any adult that was living with the child on their 1st, 5th, 8th or 12th birthday. We assigned a date of exposure that corresponded with the end date in each exposure period, so that the analysis could take account of the temporal relationship between exposure and outcome.

#### Outcome measures

We categorised READ codes (from primary care GP data) relevant to child mental health diagnoses in to four categories: (i) externalising symptoms (anti-social behaviour), (ii) internalising symptoms (stress, anxiety, depression), (iii) developmental delay (learning disabilities, attention deficit) and (iv) other (e.g. eating disorders, personality disorders). A fifth outcome of any mental health was created using the categories of externalising, internalising symptoms and other, but not developmental delay. See Additional files 11 and 12, Tables 9 and 10 for all READ codes for each category.

#### Covariates

A directed acyclic graph (DAG) [33] was drawn to visualise plausible confounding relationships and to choose a suitable set of potential confounders for analyses (see Figure 2 in Additional file 2). Based on the DAG, we adjusted for the child’s sex, young maternal age (<18 years), small-area deprivation (based on Townsend score [34], using the 2001 census for income and address), single adult household, and perinatal factors including gestational age at birth, small for gestational age, twins or triplets, maternal smoking during pregnancy, parity, birthweight, congenital abnormalities and breastfeeding at birth or at 6–8 weeks.

### Statistical analysis

We used Cox proportional hazards regression models throughout, with the outcome defined as the time to the first child mental health diagnosis or symptoms as recorded by the GP. When including demographic and perinatal variables as predictors in these models, they are entered as time-fixed covariates at birth. When the five individual ACE exposure variables are included, these are discrete and time-varying, taking the value 0 until the first exposure, and 1 thereafter.

The occurrence of missing data was low (between 0% and 3.7%) for most variables. However, breastfeeding and maternal smoking had notably higher prevalence of missing data (13.2% and 65%, respectively); organisational and administrative differences between hospitals and data collation may explain why maternal smoking has a high proportion of missing data. The subset for which data were available was large enough to fit an imputation model for these covariates with sufficient precision. Stata IC was used for statistical analyses and multiple imputation [35]. We used multiple imputation with chained equations (MICE) to account for missing data under the missing at random assumption [36]; five imputations were conducted. The imputation model included all covariates, an event indicator and the cumulative baseline hazard as described [36, 37].

Results from the Cox regression models using complete cases and MICE were similar. The imputed data set had different HR’s by around ±0.05 in the majority of estimates for any mental health, internalising, other, and developmental delay, with more variation in maternal smoking ±0.15; for the externalising outcome, the estimates were similar ±0.15, but the youngest maternal age category differed by 0.22, and 0.45 for maternal smoking. We have therefore presented only results from the multiple imputation analyses. To protect participant confidentiality, counts that were less than five are noted as “<5” and a masked total is given “~”; some counts were converted to percentages to retain information and remove disclosure risk if cells were less than five, or disclosure could occur via the use of information across multiple tables.

Univariable analyses were conducted initially to estimate unadjusted (marginal) associations between each ACE and outcomes. Following this, the models were developed in three stages for each of the five mental health diagnoses categories. First, we estimated the hazard ratios (HR) for each mental health diagnosis associated with social deprivation, sociodemographic and perinatal factors. Second, we added the ACE variables and compared the conditional HRs (cHR) from this model to the first model. Third, we fitted a model with two-way product terms between social deprivation (i.e. small-area deprivation) and each ACE in models adjusted for socio-demographic and perinatal factors. Product terms were included one at a time to explore whether small-area deprivation was a moderator of ACEs and child mental health diagnoses. We did this for the outcomes of any mental health (excluding developmental delay), and developmental delay only, as the number of observations for the other outcomes were too small.

The conditional associations between a given ACE and mental health outcome can only be given a causal interpretation if the demographic and perinatal covariates included in the model, along with the other four ACEs, are sufficient to control for all confounding between the ACE in question and the outcome. To quantify the extent to which unmeasured confounding could explain away any such estimated causal effect, E values were calculated for each ACE-outcome relationship [38]. The E value can be interpreted as the minimum strength of association on the risk ratio scale that an unmeasured confounder must have with both the exposure and the outcome, after taking into account the covariates already measured, to explain away the estimated causal effect based on the observed (conditional) exposure-outcome association. Thus, an E value of 1 indicates no “evidence for causality,” whereas higher E values represent stronger evidence. We calculate the E value based on both the estimated conditional HR and the lower limit of its 95% confidence interval.

## Results

In total 191,035 eligible children born in Wales between 1998 and 2012, listed in the Wales Electronic Cohort for Children were included. The maximum length of follow-up was just under 15 years. Depending on year of birth, 191,035 children had at least 1 year of follow-up, 181,874 had up to 5 years of follow-up, 103,089 had up to 8 years follow-up, 61,379 had up to 12 years follow-up and 20,597 were followed up to 15 years.

### Cohort statistics

Sample characteristics were consistent with national population statistics for sociodemographic, ACE and mental diagnosis variables (see Additional file 14, Table 12). In our cohort, 1073 (0.6%) infants (aged <1 year) had experienced childhood victimisation, as coded during hospital admission and 1617 (1.6%) children experienced the death of a household member by the time they were aged 4 years. For mental health, 31.3% (*n* = 56,839) and 0.7% (*n* = 1281) infants (aged <1 year) lived in a household in which an adult had a common mental disorder or a serious mental illness, respectively. In addition, 8.4% lived in a household with an adult who problematically used alcohol. All ACE occurrence percentages increased with longer duration of follow-up, aside from child victimisation.

The prevalence of any mental health diagnoses (excluding developmental delay) ranged from 0.4% (*n* = 756) for children aged 1 year to 4% (*n* = 826) for children aged 12–14 years. Externalising diagnoses were present in 0.8% (*n* = 161) of children aged 12–14 years, and internalising diagnoses were present in 2.8% (*n* = 584) of children aged 12–14 years. Furthermore, 0.2% (*n* = 370) children had been diagnosed with developmental disorder before their first birthday, and this increased to 4.2% (*n* = 866) of children aged 12–14 years.

### Cox regression models

Conditional on all other variables in Model 1 (excluding ACEs), the risk of any mental health diagnosis (excluding developmental delay) was increased among children who lived in the most deprived quintile of social deprivation (estimated cHR 1.32, CI 95% 1.16–1.51, relative to the least deprived quintile) and who had younger mothers (cHR 1.27, CI 95% 1.15–1.39). However, female children were at a reduced risk (cHR 0.79, CI 95% 0.73–0.84) as were non first-born children (cHR 0.90, CI 95% 0.84–0.98); see Table 1 for more details.

**Table 1:** Prevalence and results from Cox regressions of any mental health outcomes (excluding developmental delay). Univariable, sociodemographic and perinatal only (Model 1) and including ACEs (Model 2)

Any mental health diagnoses or symptoms (excluding developmental delay) (HR 95% CI)				
	Prevalence for those diagnosed (***n*** = 3571)	Univariable	Social deprivation, demographic and perinatal variables	ACEs adjusted for demographic and perinatal variables
**Ever any household member with an alcohol-related hospital admission**				
No	2848 (79.8%)	1.00 (ref)	-	1.00 (ref)
Yes	723 (20.3%)	1.41 (1.27–1.56)	-	1.10 (0.99–1.23)
**Ever death of any household member**				
No	~3571 (100%)	1.00 (ref)	-	1.00 (ref)
Yes	<5	1.01 (0.73–1.39)	-	0.92 (0.67–1.27)
**Household member ever had a common mental health disorder or psychosis GP code**				
No	1232 (34.5%)	1.00 (ref)	-	1.00 (ref)
Yes	2339 (65.5%)	1.74 (1.63–1.87)	-	1.63 (1.52–1.75)
**Household member ever had a serious mental illness GP code**				
No	3478 (97.4%)	1.00 (ref)	-	1.00 (ref)
Yes	93 (2.6%)	1.77 (1.30–2.41)	-	1.35 (0.99–1.84)
**Any childhood victimisation hospital admission**				
No	3471 (97.2%)	1.00 (ref)	-	1.00 (ref)
Yes	100 (2.8%)	2.40 (1.69–3.40)	-	1.90 (1.34–2.69)
**Ever in a single parent household**				
No	2054 (57.5%)	1.00 (ref)	1.00 (ref)	1.00 (ref)
Yes	1517 (42.5%)	1.13 (1.04–1.22)	1.00 (0.92–1.08)	1.03 (0.95–1.11)
**Townsend deprivation quintile at birth or in first 4 months (0.4% missing data)**				
1 (least deprived)	494 (13.8%)	1.00 (ref)	1.00 (ref)	1.00 (ref)
2	590 (16.5%)	1.18 (1.04–1.34)	1.13 (0.99 - 1.28)	1.12 (0.99–1.27)
3	675 (18.9%)	1.29 (1.15–1.46)	1.17 (1.03–1.32)	1.14 (1.01–1.29)
4	772 (21.6%)	1.42 (1.26–1.60)	1.23 (1.09–1.40)	1.19 (1.05–1.35)
5 (most deprived)	1027 (28.8%)	1.63 (1.46–1.83)	1.32 (1.16–1.51)	1.26 (1.11–1.43)
**Sex**				
Male	2059 (57.7%)	1.00 (ref)	1.00 (ref)	1.00 (ref)
Female	1512 (42.3%)	0.78 (0.73–0.84)	0.79 (0.73–0.84)	0.79 (0.74–0.85)
**Breastfeeding at birth or 6-8 weeks (20.8% missing data)**				
No	1490 (41.7%)	1.00 (ref)	1.00 (ref)	1.00 (ref)
Yes	1338 (37.5%)	0.78 (0.72–0.85)	0.88 (0.81–0.96)	0.90 (0.82–0.98)
**Maternal age at birth or at 6-8 weeks (<5 missing)**				
30–34 years	12%	0.84 (0.76–0.92)	0.89 (0.80–0.98)	0.91 (0.82–1.01)
≥35 years	23%	0.80 (0.72–0.91)	0.87 (0.77–0.99)	0.89 (0.78–1.00)
25–29 years	28%	1.00 (ref)	1.00 (ref)	1.00 (ref)
<18 years	3%	1.37 (1.12–1.69)	1.14 (0.92–1.42)	1.09 (0.88–1.36)
18–24 years	34%	1.40 (1.28–1.53)	1.27 (1.15–1.39)	1.22 (1.11–1.35)
**Gestational age at birth (4.2% missing data)**				
24≤28 weeks	11 (0.3%)	1.15 (0.60–2.22)	1.07 (0.55–2.07)	1.03 (0.53–1.99)
28≤33 weeks	47 (1.3%)	1.13 (0.84–1.52)	1.09 (0.81–1.48)	1.05 (0.74–1.43)
33≤37 weeks	246 (6.9%)	1.24 (1.08–1.43)	1.24 (1.06–1.44)	1.22 (1.05–1.42)
37–43 weeks	3116 (87.3%)	1.00 (ref)	1.00 (ref)	1.00 (ref)
**Parity (0.2% missing data)**				
0	1671 (46.8%)	1.00 (ref)	1.00 (ref)	1.00 (ref)
≥1	1892 (53.0%)	0.84 (0.79–0.91)	0.90 (0.84–0.98)	0.88 (0.82–0.95)
**Multiple births**				
No	3480 (97.5%)	1.00 (ref)	1.00 (ref)	1.00 (ref)
Yes	91 (2.5%)	0.83 (0.66–1.03)	0.79 (0.63–1.00)	0.80 (0.64–1.01)
**Small for gestational age (<10th centile) (4.9% missing data)**				
No	3027 (84.8%)	1.00 (ref)	1.00 (ref)	1.00 (ref)
Yes	369 (10.3%)	1.21 (1.08–1.36)	1.11 (0.99–1.25)	1.10 (0.97–1.24)
**Congenital anomalies**				
None	3341 (93.6%)	1.00 (ref)	1.00 (ref)	1.00 (ref)
Minor	27 (0.8%)	1.02 (0.68–1.53)	0.96 (0.64–1.45)	0.95 (0.63–1.44)
Major	203 (5.7%)	1.55 (1.34–1.80)	1.51 (1.30–1.75)	1.50 (1.29–1.74)
**Maternal cigarette smoking at booking in (71.3% missing data)**				
No	739 (20.7%)	1.00 (ref)	1.00 (ref)	1.00 (ref)
Yes	285 (8.0%)	1.41 (1.08–1.84)	1.20 (0.89–1.63)	1.19 (0.88–1.60)

The inclusion of the ACE variables slightly reduced the estimated cHRs above, but an estimated conditional association between social deprivation and child mental health diagnosis remained. Living with a household member who had a common mental disorder was conditionally associated with an increased risk of any mental health diagnosis (cHR 1.63, CI 95% 1.52–1.75). Likewise, experiencing victimisation was conditionally associated with an almost doubled risk of any mental health diagnosis (cHR 1.90, CI 95% 1.34–2.69). There was relatively weaker evidence (*p*>0.05) for a conditional association between the other ACEs (living with an adult who had alcohol problems or serious mental illness, death of a household member) and having a mental health diagnosis or symptoms in childhood. The E value for common mental health disorder was 2.15, meaning that unmeasured confounding could fully explain the estimated conditional association if there were an unmeasured confounder having a relative risk association at least as large as 2.15 with both common mental health disorder of a household member and any mental health diagnosis in the child. The corresponding E value for the lower 95% confidence limit (CL) is 2.01, meaning that unmeasured confounding would overturn the statistical significance of the estimated effect if an unmeasured confounder had relative risk associations of at least 2.01 with both exposure and outcome. The corresponding E values for victimisation was 3.21 (2.01 lower 95% CL), should be considered in the context of the estimated conditional HRs for all of the ACEs and confounders (see the right most column of Table 1), which are all less than 2. With the exception of victimisation, each of the upper 95% CLs is also less than 2. This suggests that it is somewhat implausible that unmeasured confounding fully explains the estimated effects above.

For externalising diagnoses, living with an adult who had a common mental health disorder was conditionally associated with an almost two and a half times increased risk of diagnosis (cHR 2.37, CI 95% 1.99–2.82). The presence of victimisation was conditionally associated with an increased risk of almost three and a half times (cHR 3.45, CI 95% 2.09–5.69); see Table 2 and Additional file 4, Table 2 for the full model and sociodemographic and perinatal only model (Model 1). The E value for common mental health disorder was 3.02 (2.59 for the lower 95% CL), and victimisation was 6.36 (3.60 for the lower 95% CL). Although these are higher than the E values for the previous outcome (any mental health diagnosis), the strength of evidence for causality needs partly to be calibrated against the higher estimated cHRs for the exposures and measured confounders for the externalising diagnoses outcome.

**Table 2:** Prevalence and results from Cox regressions of Externalising diagnosis or symptoms. Univariable and adjusted ACEs model (Model 2); see Additional file 4, Table 2 for sociodemographic and perinatal only (Model 1)

Externalising mental health diagnoses or symptoms (HR 95% CI)			
	Prevalence for those diagnosed (***n*** = 667)	Univariable	ACEs adjusted for demographic and perinatal variables
**Ever any household member with an alcohol-related hospital admission**			
No	499 (74.8%)	1.00 (ref)	1.00 (ref)
Yes	168 (25.2%)	1.81 (1.45–2.27)	1.14 (0.90–1.43)
**Ever death of any household member**			
No	~667 (100%)	1.00 (ref)	1.00 (ref)
Yes	<5	0.57 (0.24–1.38)	0.48 (0.20–1.16)
**Household member ever had a common mental health disorder or psychosis READ code**			
No	184 (27.6%)	1.00 (ref)	1.00 (ref)
Yes	483 (72.4%)	2.75 (2.32–3.27)	2.37 (1.99–2.82)
**Household member ever had a serious mental illness READ code**			
No	645 (96.7%)	1.00 (ref)	1.00 (ref)
Yes	22 (3.3%)	2.58 (1.42–4.68)	1.68 (0.92–3.07)
**Any childhood victimisation hospital admission**			
No	638 (95.7%)	1.00 (ref)	1.00 (ref)
Yes	29 (4.4%)	5.27 (3.21–8.67)	3.45 (2.09–5.69)

These patterns were similarly reflected in the analyses with internalising symptoms as the outcome, with the common mental health disorder ACE estimated to be conditionally associated with a less pronounced but still increased risk (cHR 1.52 CI 95% 1.39–1.65); the evidence for conditional associations with the other ACEs was weaker (*p*>0.05), see Table 3 and Additional file 5, Table 3 for the full model and sociodemographic and perinatal only model (Model 1). The E value for common mental health disorder in this analysis was 2.01 (1.82 for the lower 95% CL). Again, these should partly be interpreted in relation to the estimated cHR for this outcome, which are all lower, suggesting again that it is perhaps unlikely that unmeasured confounding could explain away the estimated effect entirely.

**Table 3:** Prevalence and results from Cox regressions of Internalising diagnosis or symptoms. Univariable and adjusted ACEs model (Model 2); see Additional file 5, Table 3 for sociodemographic and perinatal only (Model 1)

Internalising mental health diagnoses or symptoms (HR 95% CI)			
	Prevalence for those diagnosed (***n*** = 2424)	Univariable	ACEs adjusted for demographic and perinatal variables
**Ever any household member with an alcohol-related hospital admission**			
No	1969 (81.2%)	1.00 (ref)	1.00 (ref)
Yes	455 (18.8%)	1.24 (1.09–1.42)	1.04 (0.91–1.20)
**Ever death of any household member**			
No	~2424 (100%)	1.00 (ref)	1.00 (ref)
Yes	<5	1.14 (0.79–1.64)	1.08 (0.75–1.56)
**Household member ever had a common mental health disorder or psychosis READ code**			
No	869 (35.9%)	1.00 (ref)	1.00 (ref)
Yes	1555 (64.2%)	1.56 (1.44–1.70)	1.52 (1.39–1.65)
**Household member ever had a serious mental illness READ code**			
No	2376 (98.0%)	1.00 (ref)	1.00 (ref)
Yes	48 (2.0%)	0.99 (0.61–1.62)	0.80 (0.49–1.31)
**Any childhood victimisation hospital admission**			
No	2386 (98.4%)	1.00 (ref)	1.00 (ref)
Yes	38 (1.6%)	1.26 (0.70–2.29)	1.08 (0.60–1.95)

For other mental health diagnoses, alcohol admission or problem in the household was conditionally associated with increased risk (cHR 1.33 CI 95% 1.02–1.73), as was common mental health disorder (cHR 1.58 CI 95% 1.32–1.90). There was only relatively weaker evidence (*p*>0.05) for the conditional association of the other ACEs with the outcome, see Table 4 for ACEs, and Additional file 6, Table 4 for the full model and sociodemographic and perinatal only model (Model 1). Alcohol admission/problem had an E value of 1.73 (1.13 for the lower CL) and common mental health disorder had an E value of 2.09 (1.72 for the lower CL). These E values are closer in magnitude to the estimated cHRs, and thus, the evidence for causality is weaker here.

**Table 4:** Prevalence and results from Cox regressions of Other diagnosis or symptoms. Univariable and adjusted ACEs model (Model 2); see Additional file 6, Table 4 for sociodemographic and perinatal only (Model 1)

Other mental health diagnoses or symptoms (HR 95% CI)			
	Prevalence for those diagnosed (***n*** = 547)	Univariable	ACEs adjusted for demographic and perinatal variables
**Ever any household member with an alcohol-related hospital admission**			
No	432 (79.0%)	1.00 (ref)	1.00 (ref)
Yes	115 (21.0%)	1.70 (1.32–2.20)	1.33 (1.02–1.73)
**Ever death of any household member**			
No	~547 (100%)	1.00 (ref)	1.00 (ref)
Yes	<5	0.45 (0.11–1.83)	0.40 (0.10–1.64)
**Household member ever had a common mental health disorder or psychosis GP code**			
No	193 (35.3%)	1.00 (ref)	1.00 (ref)
Yes	354 (64.7%)	1.78 (1.49–2.13)	1.58 (1.32–1.90)
**Household member ever had a serious mental illness GP code**			
No	529 (96.7%)	1.00 (ref)	1.00 (ref)
Yes	18 (3.3%)	2.61 (1.35–5.04)	1.87 (0.96–3.64)
**Any childhood victimisation hospital admission**			
No	522 (95.4%)	1.00 (ref)	1.00 (ref)
Yes	25 (4.6%)	2.52 (1.13–5.64)	1.86 (0.83–4.17)

Exposure to ACEs was also conditionally associated with an increased risk of developmental delay. Alcohol problem (cHR 1.12 CI 95% 1.02–1.23), common mental health disorder (cHR 1.42 CI 95% 1.33–1.51) and victimisation (cHR 1.65 CI 95% 1.23–2.20) were all conditionally associated with an increased risk; see Table 5 for ACEs, and Additional file 7, Table 5 for the full model and sociodemographic and perinatal only model (Model 1). Common mental health disorder had an E value of 1.87 (lower CL 1.73), alcohol admission/problem was 1.49 (lower CL 1.16), and victimisation was 2.69 (lower CL 1.76). As with the previous analysis, and especially for the alcohol exposure, the evidence for causality is therefore rather weak.

**Table 5:** Prevalence and Cox regressions of Developmental Delay diagnosis. Univariable and adjusted ACE’s model (Model 2); see Additional file 7, Table 5 for sociodemographic and perinatal only (Model 1)

Developmental delay (HR 95% CI)			
	Prevalence for those diagnosed (***n*** = 4882)	Univariable	ACEs adjusted for demographic and perinatal variables
**Ever any household member with an alcohol-related hospital admission**			
No	3891 (79.7%)	1.00 (ref)	1.00 (ref)
Yes	991 (20.3%)	1.36 (1.24–1.49)	1.12 (1.02–1.23)
**Ever death of any household member**			
No	4870 (99.8%)	1.00 (ref)	1.00 (ref)
Yes	12 (0.3%)	1.12 (0.81–1.54)	1.03 (0.75–1.42)
**Household member ever had a common mental health disorder or psychosis READ code**			
No	1836 (37.6%)	1.00 (ref)	1.00 (ref)
Yes	3046 (62.4%)	1.52 (1.43–1.61)	1.42 (1.33–1.51)
**Household member ever had a serious mental illness READ code**			
No	4786 (98.0%)	1.00 (ref)	1.00 (ref)
Yes	96 (2.0%)	1.26 (0.92–1.72)	0.99 (0.72–1.35)
**Any childhood victimisation hospital admission**			
No	4736 (97.0%)	1.00 (ref)	1.00 (ref)
Yes	146 (3.0%)	2.17 (1.63–2.89)	1.65 (1.23–2.20)

To better understand the combined effects of ACEs and deprivation, three-way cross tabulations of ACEs and deprivation across the two categories of any mental health (excluding developmental delay), and developmental delay were explored. This showed that the most deprived category experienced a higher proportion of ACEs compared to the least deprived; see Additional files 8 and 9, Tables 6 and 7. We explored whether deprivation moderated the relationship between ACEs and child mental health. However, there was very little evidence of larger effect sizes for the five levels of deprivation, or a gradient, as only a single association was statistically significant between common mental health disorder and the most deprived group for developmental delay (cHR 0.81, *p*<0.05), this should not be emphasised given the issue of multiple testing; see Additional file 15, Table 13 for models. Therefore, we found insufficient evidence to conclude that the conditional association between these five ACEs and child mental health outcomes were moderated by deprivation.

## Discussion

This study has explored the effects of five individual ACEs on child mental health, going beyond cumulative measures which can obscure the relative contribution of individual adversity [10, 11, 39]. After adjusting for a number of demographic and perinatal factors, we found that living with an adult who had a common mental disorder was consistently associated with an increased risk of internalising symptoms, externalising symptoms, and developmental delay. Likewise, experiencing victimisation was conditionally associated with an increased risk of any mental health diagnosis (excluding developmental delay), developmental delay and externalising symptoms, where there was an estimated three-fold increase in the conditional rate of diagnosis. Living with an adult with alcohol-related problems or admission was a significant predictor of other mental health diagnoses and developmental delay; however, these cHRs were smaller compared to other ACEs. Serious mental illness and death of a household member had no statistically significant associations with child mental health; this may be due to the rarity of these exposures.

While the relationship between ACEs and child mental health is complex, our study aligns and builds on wider research, notably studies which examined  the association between one or two ACEs  and child mental health [10, 11]. First, we found that common mental health disorders among household members were consistently conditionally associated with all child mental health outcomes. Research on individual ACEs by Merrick et al. found that household mental illness increased depressed affect and suicide attempt. Alongside this, our findings align with a review which found that parental mood disorders increase the risk for child internalising symptoms, externalising symptoms, medical difficulties and developmental delay [40, 41]. Hence, we argue that living with an adult with a common mental health disorder can be a considerable risk factor for an array of child mental health outcomes.

Second, we found associations between alcohol problems or admissions for other mental health diagnoses (e.g. eating disorders, personality disorders) and developmental delay. In our study, it was unprecedented that there was no evidence of alcohol admissions or problems being associated with externalising symptoms. Our findings support Hussong et al. who did not find an association with internalising symptoms [15], but do not support the association with externalising symptoms [14]. Moreover, they do not support Finan et al. (2015) who found maternal alcohol use was associated with aggressive behaviour and externalising symptoms; they note that there were different mediation pathways for boys and girls through family functioning [42]. We suspect that the null result observed could be due to gender differences, the low likelihood of being diagnosed at that age, or that our measure of alcohol problems were not sufficiently sensitive to capture this.

Nevertheless, our findings can be explained by several theories, but most notably parenting, the family environment and theories of intra-generational transmission. Parenting and the family environment can be altered in situations where the parent is experiencing mental health problems or substance dependence or problems [43–45]. For instance, Davis et al. found that parents who experience depression were more likely to use punitive discipline and implement fewer household routines [46], and Smith states that these behaviours can negatively impact child wellbeing broadly, but specifically child mental health [47]. Parental mental health and substance use may also increase household conflict [43, 48, 49], which is also associated with child mental health [50].

Victimisation was also consistently associated with child mental health in this study. While only a small proportion of children in our sample was recorded as having a childhood victimisation hospital admission, our findings support that abuse is coded only in a small number of cases in hospital settings (1%) [51]. Our findings align with other research that identifies that victimisation has a profound impact on child mental health [52–54]. For instance, Nelson et al. conducted a systematic review and found that children who experienced non-sexual child maltreatment had increased mental health disorders and suicide attempts. Therefore, we further highlight the profound effect victimisation can have on child mental health diagnoses, specifically developmental delay and externalising symptoms.

Alongside ACEs, socio-demographic and perinatal aspects were also associated with the rate of diagnosis. Most notably, deprivation was consistently associated with higher rates of child mental health outcomes, which echoes Reiss [18]. Females were less likely to have a diagnosis compared to males across every outcome. This was unexpected as often females are more likely to exhibit greater mental health disorders [55]; however, we attribute this to age-related differences. Younger mothers had children with a steeper rate of diagnosis, and maternal smoking was associated with any mental health (excluding developmental delay), externalising symptoms and developmental delay; this is further evidenced in other studies, particularly for externalising symptoms [56, 57].

Alongside evaluating individual associations, our analysis explored whether deprivation could moderate the relationship between ACEs and child mental health outcomes. We found that although ACEs are socially patterned, the effects of social deprivation on child mental health are not fully explained through the five ACEs used in this analysis. We found evidence that both social deprivation and ACEs independently increase the risk of mental health diagnoses in children, with no strong evidence to reject additivity of these effects on the conditional log hazard ratio scale. However, other studies, such as Font and Maguire-Jack [58], found that socioeconomic status was a key mediator in the relationship between ACEs and adult depression. Straatmann et al. suggest ACEs mediate the association between socioeconomic status and adolescent health outcomes so there is a complex interrelationship between ACEs, socioeconomic status, and child mental health which was not fully addressed by our study [59].

Nevertheless, our research has a number of strengths. First, we do not rely on self-reported data (e.g. Felitti et al. 1998) which may be subject to poor recall [60] as the accuracy of retrospective reports of childhood events can be influenced by any number of factors, including the child’s age, memory difficulties, wanting to protect parents, a desire to deny or forget the past, or methodological research issues [61]. Second, our study adopted a unique approach compared with existing work in this area using a large number of unselected participants from a total population cohort and longitudinal follow-up over 14 years. This data permitted statistical analysis that controlled for many potentially confounding factors related to child mental health and that respected the temporal relationship between exposures and outcomes.

However, previous research has highlighted that GPs report difficulties in the recognition and detection of mental health signs and symptoms in young people compared to adult mental health, where GPs have at their disposal a checklist that helps decide on the need for a referral [62]. With regard to ACE variables, the use of ICD-10 codes to classify child victimisation has been previously found to be underestimated [63]. This is due to doctors not recognising and documenting abuse and clinical coders not being diligent in their coding; however, externalising symptoms are easier to recognise, as documented by the increased prevalence in this study [64]. Several adverse childhood events could not be measured with routine data, such as domestic violence, incarceration, and sexual and physical abuse, and parental drug use [65].

Moreover, we do not assess multiple events, cumulative scoring or chronic exposure in our study, and we encourage future research to compare these where possible. There would have been participants who required treatment for childhood mental health disorders, but for whom there was a sufficient lag between needing treatment and being diagnosed or treated who would have been unidentified in our study. While we included many potential confounders in our analyses, it is likely that the conditional associations we report still cannot be given a causal interpretation due to unmeasured confounding, for example by ethnicity. Yet further caution is required given that conditional hazard ratios present particular difficulties for causal interpretability [66]. However, as demonstrated by the calculated E values, the strength of unmeasured confounding would need to be considerable (arguably implausibly so) for most of our estimated effects to be explained away entirely.

From this, we suggest that future work should explore the association between ACEs and child mental health further, including examining factors that may mediate the relationship, such as maladaptive coping strategies for dealing with stress, and those that may moderate the relationship such as the role of protective factors. Moreover, considerable work is required to understand how socioeconomic status dynamically operates alongside ACEs, and the public health implications this has [19, 20]. Likewise, the relationship between positive childhood experiences and child mental health should be explored, given that evidence suggests that mental health is more than just the absence of adversity [67]. Furthermore, while this study investigated the impact of household mental health diagnoses on child mental health, there may be reciprocity, which should be further explored.

## Conclusions

Our findings evidence the importance of the caregiving environment and social conditions for child mental health. They show conditional associations between sociodemographic factors, perinatal aspects, and ACEs specifically common mental health disorders and victimisation, with child mental health outcomes. However, we did not find evidence for moderation of the effect of ACEs by deprivation. Our findings offer further evidence to support the need for structural interventions, reducing modifiable socioeconomic inequalities, and the early identification and evidence-based intervention for all children who have experienced ACEs [68, 69]; models such as the Building Community Resilience (BCR) have been viewed as positive [69]; however, interventions using ACE screening require further research to establish their effectiveness [70].

## Supplementary Information

**Additional file 1: Figure 1.** Participant Selection.

**Additional file 2: Figure 2.** Directed acyclic graph of exposure to ACEs and child mental health outcome at 8 years.

**Additional file 3: Table 1.** Sociodemographic and perinatal characteristics in each exposure.

**Additional file 4: Table 2.** Externalising Symptoms (Table 2 continued for confounders) prevalence, univariable analyses, sociodemographic and perinatal aspects, and ACEs Cox regression.

**Additional file 5: Table 3.** Internalising Symptoms (Table 3 continued for confounders) prevalence, univariable analyses, sociodemographic and perinatal aspects, and ACEs Cox regression.

**Additional file 6: Table 4.** Other diagnosis or symptom (Table 4 continued for confounders) prevalence, univariable analyses, sociodemographic and perinatal aspects, and ACEs Cox regression.

**Additional file 7: Table 5.** Developmental delay (Table 5 continued for confounders) prevalence, univariable analyses, sociodemographic and perinatal aspects, and ACEs Cox regression.

**Additional file 8: Table 6.** Three-way cross-tabulation of ACEs, Deprivation and Any Mental Health (excluding developmental delay).

**Additional file 9: Table 7.** Three-way cross-tabulation of ACEs, Deprivation and Developmental Delay.

**Additional file 10: Table 8.** Adult Mental Health codes for Common Mental Disorder (CMD) and Serious Mental Illness (SMI).

**Additional file 11: Table 9.** Codes for Externalising and Internalising categories.

**Additional file 12: Table 10.** Codes for Other and Developmental delay.

**Additional file 13: Table 11.** ICD-10 codes for victimisation, taken from Lee, Gonzalez-Izquierod, and Gilbert (2012) [27].

**Additional file 14: Table 12.** Table of national population statistics.

**Additional file 15: Table 13.** Moderation analysis for ACEs, deprivation, and child mental health.

## Abbreviations

ACEs: Adverse childhood experiences
cHR: Conditional HRs
CI: Confidence interval
CL: Confidence limit
DAG: Directed acyclic graph
GP: General practice
HR: Hazard ratios
MICE: Multiple imputation with chained equations
NHS: National Health Service
NWIS: NHS Wales Informatics Service
RALFs: Residential Anonymised Linking Field
SAIL: Secure Anonymised Information Linkage
SDQ: Strengths and Difficulties Questionnaire
WECC: Wales Electronic Cohort for Children
