# Supplementary material for: Adverse childhood experiences and child mental health: an electronic birth cohort study
Source: BMC Med. 2021 Aug 6;19:172. doi: 10.1186/s12916-021-02045-x (PMC8344166; doi:10.1186/s12916-021-02045-x)
Supplement: Supplementary file 1 — Additional file 1: Figure 1. Participant Selection. [file 12916_2021_2045_MOESM1_ESM.docx]

**Additional File 1: Figure 1 - Participant Selection**
