# Supplementary material for: Adverse childhood experiences and child mental health: an electronic birth cohort study
Source: BMC Med. 2021 Aug 6;19:172. doi: 10.1186/s12916-021-02045-x (PMC8344166; doi:10.1186/s12916-021-02045-x)

**Additional File 2: Figure 2 - Directed acyclic graph of exposure to ACEs and child mental health outcome at 8 years.**


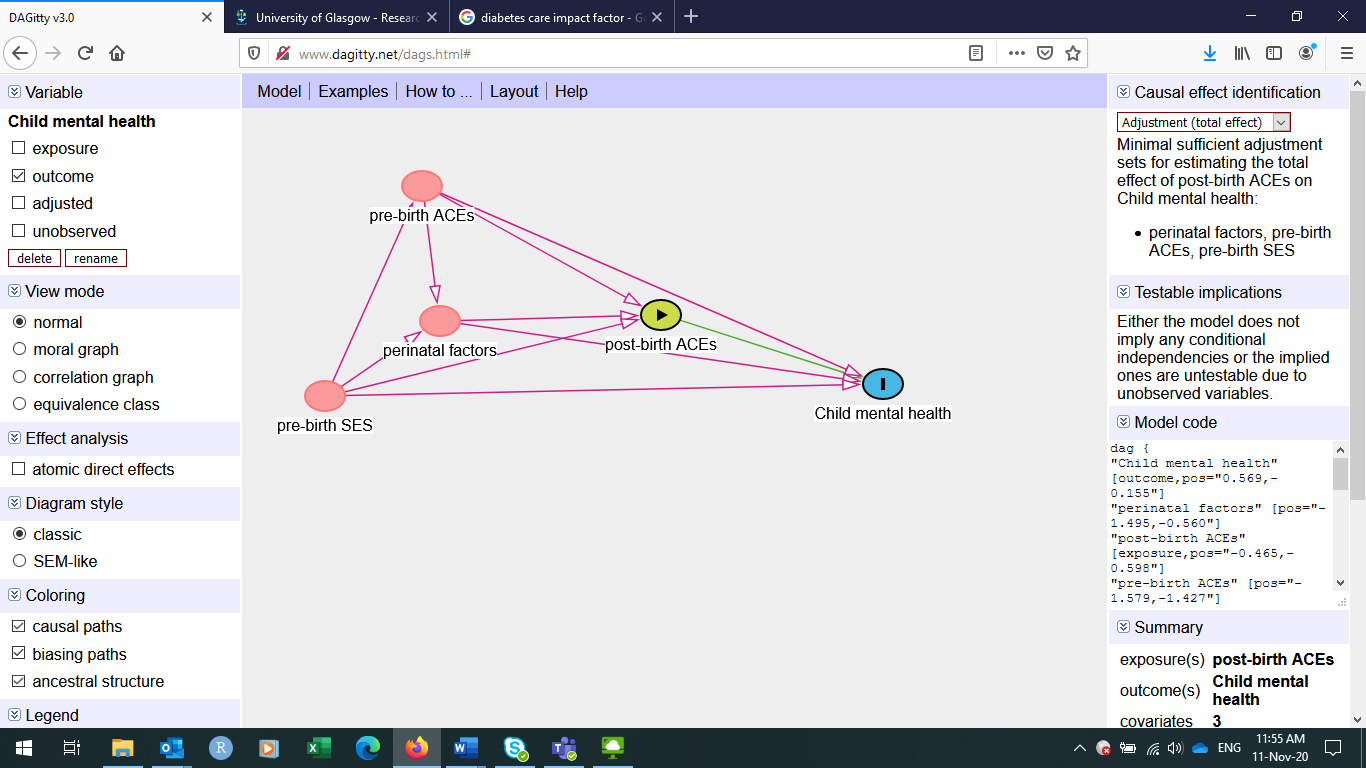

Supplement: Supplementary file 2 — Additional file 2: Figure 2. Directed acyclic graph of exposure to ACEs and child mental health outcome at 8 years. [file 12916_2021_2045_MOESM2_ESM.docx]
