# Supplementary material for: Adverse childhood experiences and child mental health: an electronic birth cohort study
Source: BMC Med. 2021 Aug 6;19:172. doi: 10.1186/s12916-021-02045-x (PMC8344166; doi:10.1186/s12916-021-02045-x)
Supplement: Supplementary file 3 — Additional file 3: Table 1. Sociodemographic and perinatal characteristics in each exposure. [file 12916_2021_2045_MOESM3_ESM.docx]

**Additional File 3: Table 1 - Sociodemographic and perinatal characteristics in each exposure**

|  | **Total**  **(n = 191 035)** | **Ever lived with household members with a common mental disorder**  **(n = 94404)** | **Ever lived with household members with a serious mental illness**  **(n = 2917)** | **Ever lived with household members with alcohol-related hospital admission or GP record**  **(n = 29324)** | **Ever admitted to hospital for childhood victimisation**  **(n = 2099)** | **Death of any household members**  **(n=7878)** |
| --- | --- | --- | --- | --- | --- | --- |
| **Townsend deprivation quintile at birth or in first 4 months** | | | | | | |
| 1 (least deprived) | 33 704 (17.6%) | 14 349  (15.2%) | 11% | 2911 (9.9%) | 192 (9.2%) | 1107 (14.1%) |
| 2 | 34 671 (18.1%) | 15 615  (16.5%) | 14% | 4067 (13.9%) | 317 (15.1%) | 1421 (18.0%) |
| 3 | 37 245 (19.5%) | 18 252  (19.3%) | 17% | 5362 (18.3%) | 344 (16.4%) | 1513 (19.2%) |
| 4 | 39 472 (20.7%) | 20 634  (21.9%) | 24% | 6906 (23.6%) | 417 (19.9%) | 1728 (21.9%) |
| 5 (most deprived) | 45 595 (23.9%) | 25 433  (26.9%) | 34% | 10 030 (34.3%) | 824 (39.4%) | 2086 (26.5%) |
| Missing data | 348 (0.2%) | 121 (0.1%) | <5 | 48 (0.2%) | 5 (0.2%) | 23 (0.3%) |
| **Sex** | | | | | | |
| Male | 98 302 (51.5%) | 48 529  (51.4%) | 1512 (51.8%) | 15 012 (51.2%) | 1087 (51.8%) | 3833 (48.7%) |
| Female | 92 733 (48.5%) | 45 875  (48.6%) | 1405 (48.2%) | 14 312 (48.8%) | 1012 (48.2%) | 4045 (51.3%) |
| **Breastfeeding at birth or at 6-8 weeks** | | | | | | |
| No | 73 577 (38.5%) | 40 970  (43.4%) | 1350 (46.3%) | 14 017 (47.8%) | 1064 (50.7%) | 3089 (39.2%) |
| Yes | 92 321 (48.3%) | 41 347  (43.8%) | 1211 (41.5%) | 11 372 (38.8%) | 761 (36.3%) | 3372 (42.8%) |
| Missing data | 25 137 (13.2%) | 12 087  (12.8%) | 356 (12.2%) | 3935 (13.4%) | 274 (13.1%) | 1417 (18.0%) |
| **Maternal age at childbirth** | | | | | | |
| <18 years | 4339 (2.3%) | 2669 (2.8%) | 4% | 1485 (5.1%) | 124 (5.9%) | 4% |
| 18-24 years | 49 869 (26.1%) | 28 190  (29.9%) | 35% | 11 168 (38.1%) | 821 (39.1%) | 30% |
| 25-29 years | 53 278 (27.9%) | 26 172  (27.7%) | 26% | 7359 (25.1%) | 514 (24.5%) | 25% |
| 30-34 years | 51 952 (27.2%) | 23 076  (24.4%) | 20% | 5714 (19.5%) | 377 (18.0%) | 24% |
| ≥35 years | 31 512 (16.5%) | 14 261  (15.1%) | 15% | 3582 (12.2%) | 256 (12.2%) | 17% |
| Missing data | 85 (0.0%) | 36  (0.0%) | <5 | 16 (0.1%) | 7 (0.3%) | <5 |
| **Gestational age at birth** | | | | | | |
| 24-<28 weeks | 510 (0.3%) | 270 (0.3%) | 12 (0.4%) | 87 (0.3%) | 19 (0.9%) | 19 (0.2%) |
| 28-<33 weeks | 2325 (1.2%) | 1257 (1.3%) | 43 (1.5%) | 382 (1.3%) | 52 (2.5%) | 117 (1.5%) |
| 33-<37 weeks | 10514 (5.5%) | 5473 (5.8%) | 173 (5.9%) | 1753 (6.0%) | 190 (9.1%) | 521 (6.6%) |
| 37-43 weeks | 17 1564 (89.8%) | 84 324 (89.3%) | 2582 (88.5%) | 26 065 (88.9%) | 1783 (84.9%) | 6851 (87.0%) |
| Missing data | 6122 (3.2%) | 3080 (3.3%) | 107 (3.7%) | 1037 (3.5%) | 55 (2.6%) | 370 (4.7%) |
| **Small for gestational age (<10^th^ centile)** | | | | | | |
| No | 166 552 (87.2%) | 81 959 (86.8%) | 2446 (83.9%) | 24 793 (84.5%) | 1690 (80.5%) | 6620 (84.0%) |
| Yes | 17 356 (9.1%) | 8854 (9.4%) | 343 (11.8%) | 3362 (11.5%) | 337 (16.1%) | 838 (10.6%) |
| Missing data | 7127 (3.7%) | 3591 (3.8%) | 128 (4.4%) | 1169 (4.0%) | 72 (3.4%) | 420 (5.3%) |
| **Parity** | | | | | | |
| 0 | 82 703 (43.3%) | 38 543 (40.8%) | 41% | 12 383 (42.2%) | 898 (42.8%) | 3711 (47.1%) |
| ≥1 | 108 005 (56.5%) | 55 713 (59.0%) | 59% | 16 892 (57.6%) | 1190 (56.7%) | 4151 (52.7%) |
| Missing data | 327 (0.2%) | 148 (0.2%) | <5 | 49 (0.2%) | 11 (0.5%) | 16 (0.2%) |
| **Multiple births (e.g., twins)** | | | | | | |
| No or missing data | 185 289 (97.0%) | 91 545 (97.0%) | 2835 (97.2%) | 28 602 (97.5%) | 2044 (97.4%) | 7614 (96.6%) |
| Yes | 5746 (3.0%) | 2859 (3.0%) | 82 (2.8%) | 722 (2.5%) | 55 (2.6%) | 264 (3.4%) |
| **Birthweight** | | | | | | |
| Low (<2500g) | 12 052 (6.3%) | 6382 (6.8%) | 203 (7.0%) | 2295 (7.8%) | 292 (13.9%) | 621 (7.9%) |
| Normal (2500-3999g) | 149 721 (78.4%) | 73 788 (78.2%) | 2303 (79.0%) | 22 991 (78.4%) | 1569 (74.7%) | 6043 (76.7%) |
| High (≥4000g) | 22 135 (11.6%) | 10 643 (11.3%) | 283 (9.7%) | 2869 (9.8%) | 166 (7.9%) | 794 (10.1%) |
| Missing data | 7127 (3.7%) | 3591 (3.8%) | 128 (4.4%) | 1169 (4.0%) | 72 (3.4%) | 420 (5.3%) |
| **Congenital abnormalities** | | | | | | |
| None | 182 774 (95.7%) | 90 191 (95.5%) | 2782 (95.4%) | 28 030 (95.6%) | 1902 (90.6%) | 7513 (95.4%) |
| Minor | 1297 (0.7%) | 686 (0.7%) | 23 (0.8%) | 212 (0.7%) | 23 (1.1%) | 52 (0.7%) |
| Major | 6964 (3.6%) | 3527 (3.7%) | 112 (3.8%) | 1082 (3.7%) | 174 (8.3%) | 313 (4.0%) |
| **Maternal smoking at booking in for birth** | | | | | | |
| No | 52 478 (27.5%) | 23136 (24.5%) | 595 (20.4%) | 6299 (21.5%) | 437 (20.8%) | 2015 (25.6%) |
| Yes | 14 295 (7.5%) | 8247 (8.7%) | 336 (11.5%) | 3697 (12.6%) | 291 (13.9%) | 739 (9.4%) |
| Missing data | 124 262 (65.0%) | 63021 (66.8%) | 1986 (68.1%) | 19 328 (65.9%) | 1371 (65.3%) | 5124 (65.0%) |
