# Supplementary material for: Adverse childhood experiences and child mental health: an electronic birth cohort study
Source: BMC Med. 2021 Aug 6;19:172. doi: 10.1186/s12916-021-02045-x (PMC8344166; doi:10.1186/s12916-021-02045-x)
Supplement: Supplementary file 4 — Additional file 4: Table 2. Externalising Symptoms (Table 2 continued for confounders) prevalence, univariable analyses, sociodemographic and perinatal aspects, and ACEs Cox regression. [file 12916_2021_2045_MOESM4_ESM.docx]

**Additional File 4: Table 2 - Externalising Symptoms (Table 2 continued for confounders) prevalence, univariable analyses, sociodemographic and perinatal aspects, and ACEs Cox regression**

| **Externalising (HR 95% CI)** | | | | |
| --- | --- | --- | --- | --- |
|  | **Prevalence for those diagnosed (n=667)** | **Univariable** | **Demographic and Perinatal variables** | **ACEs adjusted for demographic and perinatal variables** |
| **Ever in a single parent household** | | | | |
| No | 290 (43.5%) | 1.00 (ref) | 1.00 (ref) | 1.00 (ref) |
| Yes | 377 (56.5%) | 1.79 (1.51 – 2.12) | 1.41 (1.18 - 1.68) | 1.48 (1.24 - 1.76) |
| **Townsend deprivation quintile at birth or in first 4 months (<5 missing data)** | | | | |
| 1 (least deprived) | 10% | 1.00 (ref) | 1.00 (ref) | 1.00 (ref) |
| 2 | 12% | 1.13 (0.80 – 1.60) | 0.99 (0.70 - 1.40) | 0.97 (0.69 - 1.37) |
| 3 | 17% | 1.53 (1.11 – 2.10) | 1.17 (0.84 - 1.62) | 1.12 (0.81 - 1.55) |
| 4 | 22% | 1.91 (1.40 – 2.59) | 1.32 (0.95 - 1.83) | 1.23 (0.89 - 1.70) |
| 5 (most deprived) | 38% | 2.83 (2.13 – 3.77) | 1.62 (1.16 - 2.25) | 1.48 (1.06 - 2.05) |
| **Sex** | | | | |
| Male | 481 (72.1%) | 1.00 (ref) | 1.00 (ref) | 1.00 (ref) |
| Female | 186 (27.9%) | 0.40 (0.33 – 0.48) | 0.40 (0.34 - 0.49) | 0.41 (0.34 - 0.49) |
| **Breastfeeding at birth or 6-8 weeks (23.5% missing data)** | | | | |
| No | 295 (44.2%) | 1.00 (ref) | 1.00 (ref) | 1.00 (ref) |
| Yes | 215 (32.2%) | 0.64 (0.50 – 0.81) | 0.91 (0.67 - 1.24) | 0.94 (0.69 - 1.28) |
| **Maternal age at birth or at 6-8 weeks** | | | | |
| 30-34 years | 124 (18.6%) | 0.83 (0.64 – 1.06) | 0.95 (0.74 - 1.23) | 1.00 (0.77 - 1.29) |
| ≥35 years | 62 (9.3%) | 0.71 (0.52 – 0.98) | 0.87 (0.63 - 1.21) | 0.90 (0.65 - 1.25) |
| 25-29 years | 153 (22.9%) | 1.00 (ref) | 1.00 (ref) | 1.00 (ref) |
| <18 years | 34 (5.1%) | 2.55 (1.69 – 3.83) | 1.86 (1.20 - 2.88) | 1.72 (1.11 - 2.67) |
| 18-24 years | 294 (44.1%) | 2.09 (1.69 – 2.59) | 1.66 (1.32 - 2.08) | 1.56 (1.24 - 1.96) |
| **Gestational age at birth (5% missing data)** | | | | |
| 24-<28 weeks | <5 | 1.44 (0.36 – 5.78) | 1.20 (0.30 - 4.85) | 1.09 (0.27 - 4.43) |
| 28-<33 weeks | 2% | 1.88 (1.09 – 3.27) | 1.65 (0.94 - 2.89) | 1.55 (0.89 - 2.72) |
| 33-<37 weeks | 6% | 0.97 (0.65 – 1.45) | 0.95 (0.62 - 1.46) | 0.92 (0.60 - 1.40) |
| 37-43 weeks | 87% | 1.00 (ref) | 1.00 (ref) | 1.00 (ref) |
| **Parity** | | | | |
| 0 | 45% | 1.00 (ref) | 1.00 (ref) | 1.00 (ref) |
| ≥1 | 55% | 0.90 (0.76 – 1.06) | 1.06 (0.88 - 1.27) | 1.01 (0.84 -1.21) |
| **Multiple births** | | | | |
| No | 652 (97.8%) | 1.00 (ref) | 1.00 (ref) | 1.00 (ref) |
| Yes | 15 (2.2%) | 0.69 (0.39 – 1.21) | 0.75 (0.41 - 1.36) | 0.76 (0.41 - 1.38) |
| **Small for gestational age (<10th centile)** | | | | |
| No | 547 (82.0%) | 1.00 (ref) | 1.00 (ref) | 1.00 (ref) |
| Yes | 83 (12.4%) | 1.55 (1.21 – 1.98) | 1.24 (0.95 - 1.62) | 1.22 (0.93 - 1.59) |
| **Congenital anomalies** | | | | |
| None | 626 (93.9%) | 1.00 (ref) | 1.00 (ref) | 1.00 (ref) |
| Minor | 6 (0.9%) | 1.48 (0.66 – 3.32) | 1.23 (0.55 - 2.77) | 1.21 (0.54 - 2.72) |
| Major | 35 (5.2%) | 1.28 (0.87 – 1.87) | 1.20 (0.82 - 1.76) | 1.16 (0.79 - 1.71) |
| **Maternal cigarette smoking at booking in** | | | | |
| No | 97 (14.5%) | 1.00 (ref) | 1.00 (ref) | 1.00 (ref) |
| Yes | 71 (10.6%) | 2.74 (1.42 – 5.30) | 2.02 (0.93 - 4.38) | 1.96 (0.91 - 4.25) |
