# Supplementary material for: Adverse childhood experiences and child mental health: an electronic birth cohort study
Source: BMC Med. 2021 Aug 6;19:172. doi: 10.1186/s12916-021-02045-x (PMC8344166; doi:10.1186/s12916-021-02045-x)
Supplement: Supplementary file 5 — Additional file 5: Table 3. Internalising Symptoms (Table 3 continued for confounders) prevalence, univariable analyses, sociodemographic and perinatal aspects, and ACEs Cox regression. [file 12916_2021_2045_MOESM5_ESM.docx]

**Additional File 5: Table 3 - Internalising Symptoms (Table 3 continued for confounders) prevalence, univariable analyses, sociodemographic and perinatal aspects, and ACEs Cox regression**

| **Internalising (HR 95% CI)** | | | | |
| --- | --- | --- | --- | --- |
|  | **Prevalence for those diagnosed (n=2424)** | **Univariable** | **Demographic and Perinatal variables** | **ACEs adjusted for demographic and perinatal variables** |
| **Ever in a single parent household** | | | | |
| No | 968 (39.9%) | 1.00 (ref) | 1.00 (ref) | 1.00 (ref) |
| Yes | 1456 (60.1%) | 1.02 (0.93 – 1.12) | 0.92 (0.84 - 1.02) | 0.95 (0.86 – 1.04) |
| **Townsend deprivation quintile at birth or in first 4 months (0.4% missing data)** | | | | |
| 1 (least deprived) | 372 (15.3%) | 1.00 (ref) | 1.00 (ref) | 1.00 (ref) |
| 2 | 433 (17.9%) | 1.13 (0.98 – 1.30) | 1.09 (0.95 - 1.27) | 1.09 (0.94 – 1.26) |
| 3 | 452 (18.6%) | 1.14 (0.99 – 1.32) | 1.07 (0.92 - 1.24) | 1.05 (0.91 – 1.22) |
| 4 | 521 (21.5%) | 0.94 (0.86 – 1.02) | 1.13 (0.98 - 1.31) | 1.11 (0.96 – 1.28) |
| 5 (most deprived) | 637 (26.3%) | 0.85 (0.77 – 0.95) | 1.18 (1.02 - 1.38) | 1.14 (0.98 – 1.33) |
| **Sex** | | | | |
| Male | 1287 (53.1%) | 1.00 (ref) | 1.00 (ref) | 1.00 (ref) |
| Female | 1137 (46.9%) | 0.94 (0.86 – 1.02) | 0.94 (0.87 - 1.02) | 0.94 (0.87 – 1.02) |
| **Breastfeeding at birth or 6-8 weeks (20.6% missing data)** | | | | |
| No | 971 (40.1%) | 1.00 (ref) | 1.00 (ref) | 1.00 (ref) |
| Yes | 953 (39.3%) | 0.85 (0.77 – 0.95) | 0.91 (0.82 - 1.02) | 0.93 (0.83 – 1.03) |
| **Maternal age at birth or at 6-8 weeks (<5 missing data)** | | | | |
| 30-34 years | 24% | 0.85 (0.76 – 0.96) | 0.89 (0.79 - 1.00) | 0.91 (0.80 – 1.02) |
| ≥35 years | 13% | 0.81 (0.70 – 0.93) | 0.87 (0.75 - 1.00) | 0.87 (0.76 – 1.01) |
| 25-29 years | 29% | 1.00 (ref) | 1.00 (ref) | 1.00 (ref) |
| <18 years | 3% | 1.16 (0.89 – 1.51) | 0.96 (0.73 - 1.27) | 0.93 (0.71 – 1.23) |
| 18-24 years | 32% | 1.30 (1.17 – 1.45) | 1.20 (1.07 - 1.35) | 1.17 (1.04 – 1.31) |
| **Gestational age at birth (4.2% missing data)** | | | | |
| 24-<28 weeks | 11 (0.3%) | 0.92 (0.38 – 2.20) | 0.89 (0.37 - 2.15) | 0.87 (0.36 – 2.12) |
| 28-<33 weeks | 47 (1.3%) | 0.85 (0.56 – 1.28) | 0.85 (0.56 - 1.30) | 0.83 (0.55 – 1.27) |
| 33-<37 weeks | 246 (6.9%) | 1.29 (1.10 – 1.52) | 1.32 (1.11 - 1.56) | 1.30 (1.10 – 1.54) |
| 37-43 weeks | 3116 (87.3%) | 1.00 (ref) | 1.00 (ref) | 1.00 (ref) |
| **Parity (0.2% missing data)** | | | | |
| 0 | 1193 (49.2%) | 1.00 (ref) | 1.00 (ref) | 1.00 (ref) |
| ≥1 | 1226 (50.6%) | 0.77 (0.71 – 0.84) | 0.81 (0.74 - 0.88) | 0.79 (0.72 – 0.86) |
| **Multiple births** | | | | |
| No | 2365 (97.6%) | 1.00 (ref) | 1.00 (ref) | 1.00 (ref) |
| Yes | 59 (2.4%) | 0.79 (0.60 – 1.03) | 0.76 (0.57 - 1.01) | 0.76 (0.57 – 1.01) |
| **Small for gestational age (<10th centile)** | | | | |
| No | 2082 (85.9%) | 1.00 (ref) | 1.00 (ref) | 1.00 (ref) |
| Yes | 220 (9.1%) | 1.06 (0.92 – 1.22) | 0.99 (0.85 - 1.15) | 0.98 (0.84 – 1.14) |
| **Congenital anomalies** | | | | |
| None | 2281 (94.1%) | 1.00 (ref) | 1.00 (ref) | 1.00 (ref) |
| Minor | 17 (0.7%) | 0.89 (0.53 – 1.50) | 0.87 (0.52 - 1.48) | 0.87 (0.51 – 1.47) |
| Major | 126 (5.2%) | 1.43 (1.19 – 1.72) | 1.42 (1.18 - 1.70) | 1.42 (1.18 – 1.70) |
| **Maternal cigarette smoking at booking in** | | | | |
| No | 537 (22.2%) | 1.00 (ref) | 1.00 (ref) | 1.00 (ref) |
| Yes | 179 (7.4%) | 1.24 (0.90 – 1.70) | 1.12 (0.79 - 1.59) | 1.11 (0.78 – 1.58) |
