# Supplementary material for: Adverse childhood experiences and child mental health: an electronic birth cohort study
Source: BMC Med. 2021 Aug 6;19:172. doi: 10.1186/s12916-021-02045-x (PMC8344166; doi:10.1186/s12916-021-02045-x)
Supplement: Supplementary file 6 — Additional file 6: Table 4. Other diagnosis or symptom (Table 4 continued for confounders) prevalence, univariable analyses, sociodemographic and perinatal aspects, and ACEs Cox regression. [file 12916_2021_2045_MOESM6_ESM.docx]

**Additional File 6: Table 4 - Other diagnosis or symptom (Table 4 continued for confounders) prevalence, univariable analyses, sociodemographic and perinatal aspects, and ACEs Cox regression**

| **Other (HR 95% CI)** | | | | |
| --- | --- | --- | --- | --- |
|  | **Prevalence for those diagnosed**  **(n =547)** | **Univariable** | **Demographic and Perinatal variables** | **ACEs adjusted for demographic and perinatal variables** |
| **Ever in a single parent household** | | | | |
| No | 339 (62.0%) | 1.00 (ref) | 1.00 (ref) | 1.00 (ref) |
| Yes | 208 (38.0%) | 0.95 (0.77 – 1.17) | 0.85 (0.69 - 1.05) | 0.89 (0.72 – 1.10) |
| **Townsend deprivation quintile at birth or in first 4 months (<5 missing data)** | | | | |
| 1 (least deprived) | 12% | 1.00 (ref) | 1.00 (ref) | 1.00 (ref) |
| 2 | 15% | 1.31 (0.93 – 1.85) | 1.27 (0.90 - 1.79) | 1.26 (0.89 – 1.77) |
| 3 | 22% | 1.76 (1.27 – 2.42) | 1.61 (1.16 - 2.23) | 1.57 (1.13 – 2.17) |
| 4 | 21% | 1.73 (1.25 – 2.38) | 1.54 (1.10 - 2.14) | 1.47 (1.05 – 2.05) |
| 5 (most deprived) | 30% | 1.97 (1.45 – 2.68) | 1.66 (1.17 - 2.34) | 1.55 (1.10 – 2.19) |
| **Sex** | | | | |
| Male | 340 (62.2%) | 1.00 (ref) | 1.00 (ref) | 1.00 (ref) |
| Female | 207 (37.8%) | 0.66 (0.55 – 0.79) | 0.67 (0.56 - 0.80) | 0.67 (0.56 – 0.80) |
| **Breastfeeding at birth or 6-8 weeks (19.4% missing data)** | | | | |
| No | 245 (44.8%) | 1.00 (ref) | 1.00 (ref) | 1.00 (ref) |
| Yes | 196 (35.8%) | 0.66 (0.55 – 0.80) | 0.75 (0.61 - 0.92) | 0.77 (0.63 – 0.94) |
| **Maternal age at birth or at 6-8 weeks** | | | | |
| 30-34 years | 120 (21.9%) | 0.78 (0.61 – 1.00) | 0.81 (0.63 - 1.04) | 0.83 (0.64 – 1.07) |
| ≥35 years | 80 (14.6%) | 0.92 (0.70 – 1.22) | 0.96 (0.72 - 1.29) | 0.98 (0.73 – 1.31) |
| 25-29 years | 155 (28.3%) | 1.00 (ref) | 1.00 (ref) | 1.00 (ref) |
| <18 years | 19 (3.5%) | 1.38 (0.82 – 2.32) | 1.33 (0.77 - 2.31) | 1.24 (0.72 – 2.15) |
| 18-24 years | 173 (31.6%) | 1.21 (0.96 – 1.52) | 1.16 (0.91 - 1.49) | 1.11 (0.87 – 1.42) |
| **Gestational age at birth (3.0% missing data, rounded % or SDC)** | | | | |
| 24-<28 weeks | <5 | 1.70 (0.42 – 6.82) | 1.36 (0.33 - 5.52) | 1.28 (0.31 – 5.20) |
| 28-<33 weeks | ~5% | 1.37 (0.68 – 2.78) | 1.17 (0.56 - 2.42) | 1.12 (0.54 – 2.31) |
| 33-<37 weeks | ~10% | 1.24 (0.87 – 1.76) | 1.14 (0.79 - 1.65) | 1.12 (0.77 – 1.61) |
| 37-43 weeks | ~90% | 1.00 (ref) | 1.00 (ref) | 1.00 (ref) |
| **Parity (<5 missing data)** | | | | |
| 0 | 41% | 1.00 (ref) | 1.00 (ref) | 1.00 (ref) |
| ≥1 | 59% | 1.12 (0.94 – 1.34) | 1.19 (0.97 - 1.45) | 1.15 (0.95 – 1.41) |
| **Multiple births (e.g. twins)** | | | | |
| No | 528 (96.5%) | 1.00 (ref) | 1.00 (ref) | 1.00 (ref) |
| Yes | 19 (3.5%) | 1.21 (0.75 – 1.93) | 1.10 (0.66 - 1.83) | 1.12 (0.67 – 1.86) |
| **Small for gestational age (<10th centile) (2.9% missing data)** | | | | |
| No | 466 (85.2%) | 1.00 (ref) | 1.00 (ref) | 1.00 (ref) |
| Yes | 65 (11.9%) | 1.37 (1.04 – 1.81) | 1.30 (0.96 - 1.75) | 1.28 (0.95 – 1.72) |
| **Congenital anomalies** | | | | |
| None | 491 (89.8%) | 1.00 (ref) | 1.00 (ref) | 1.00 (ref) |
| Minor | 5 (0.9%) | 1.22 (0.45 – 3.25) | 1.11 (0.41 - 2.97) | 1.10 (0.41 – 2.95) |
| Major | 51 (9.3%) | 2.73 (2.03 – 3.68) | 2.57 (1.91 - 3.48) | 2.56 (1.89 – 3.45) |
| **Maternal cigarette smoking at booking in (71.5% missing data)** | | | | |
| No | 118 (21.6%) | 1.00 (ref) | 1.00 (ref) | 1.00 (ref) |
| Yes | 38 (6.9%) | 1.19 (0.58 – 2.47) | 0.97 (0.43 - 2.19) | 0.95 (0.42 – 2.15) |
