# Supplementary material for: Adverse childhood experiences and child mental health: an electronic birth cohort study
Source: BMC Med. 2021 Aug 6;19:172. doi: 10.1186/s12916-021-02045-x (PMC8344166; doi:10.1186/s12916-021-02045-x)
Supplement: Supplementary file 7 — Additional file 7: Table 5. Developmental delay (Table 5 continued for confounders) prevalence, univariable analyses, sociodemographic and perinatal aspects, and ACEs Cox regression. [file 12916_2021_2045_MOESM7_ESM.docx]

**Additional File 7: Table 5 - Developmental delay (Table 5 continued for confounders) prevalence, univariable analyses, sociodemographic and perinatal aspects, and ACEs Cox regression**

| **Developmental Delay (HR 95% CI)** | | | | |
| --- | --- | --- | --- | --- |
|  | **Prevalence for those diagnosed**  **(n =4882)** | **Univariable** | **Demographic and Perinatal variables** | **ACEs adjusted for demographic and perinatal variables** |
| **Ever in a single parent household** | | | | |
| No | 2894 (59.3%) | 1.00 (ref) | 1.00 (ref) | 1.00 (ref) |
| Yes | 1988 (40.7%) | 1.10 (1.03 – 1.18) | 1.02 (0.95 - 1.09) | 1.04 (0.98 – 1.12) |
| **Townsend deprivation quintile at birth or in first 4 months (0.3% missing data)** | | | | |
| 1 (least deprived) | 687 (14.1%) | 1.00 (ref) | 1.00 (ref) | 1.00 (ref) |
| 2 | 790 (16.2%) | 1.11 (1.00 – 1.23) | 1.08 (0.97 - 1.20) | 1.08 (0.97 – 1.20) |
| 3 | 954 (19.5%) | 1.26 (1.14 – 1.39) | 1.17 (1.05 - 1.29) | 1.15 (1.04 – 1.27) |
| 4 | 1020 (20.9%) | 1.31 (1.19 – 1.45) | 1.19 (1.07 - 1.31) | 1.15 (1.04 – 1.28) |
| 5 (most deprived) | 1418 (29.0%) | 1.57 (1.43 – 1.72) | 1.32 (1.19 - 1.47) | 1.27 (1.15 – 1.41) |
| **Sex** | | | | |
| Male | 3494 (71.6%) | 1.00 (ref) | 1.00 (ref) | 1.00 (ref) |
| Female | 1388 (28.4%) | 0.43 (0.40 – 0.46) | 0.44 (0.41 - 0.47) | 0.44 (0.41 – 0.47) |
| **Breastfeeding at birth or 6-8 weeks (19.0% missing data)** | | | | |
| No | 2091 (42.8%) | 1.00 (ref) | 1.00 (ref) | 1.00 (ref) |
| Yes | 1862 (38.1%) | 0.76 (0.72 – 0.81) | 0.86 (0.80 - 0.92) | 0.87 (0.81 – 0.93) |
| **Maternal age at birth or at 6-8 weeks (<5 missing data)** | | | | |
| 30-34 years | 24% | 0.95 (0.87 – 1.03) | 0.99 (0.91 - 1.08) | 1.01 (0.93 – 1.09) |
| ≥35 years | 16% | 1.05 (0.96 – 1.15) | 1.10 (1.00 - 1.21) | 1.12 (1.01 – 1.23) |
| 25-29 years | 26% | 1.00 (ref) | 1.00 (ref) | 1.00 (ref) |
| <18 years | 3% | 1.25 (1.04 – 1.51) | 1.12 (0.92 - 1.36) | 1.08 (0.89 - 1.31) |
| 18-24 years | 32% | 1.30 (1.20 – 1.41) | 1.22 (1.12 - 1.32) | 1.19 (1.09 – 1.29) |
| **Gestational age at birth (5.0% missing data)** | | | | |
| 24-<28 weeks | 49 (1.0%) | 4.58 (3.42 – 6.12) | 3.66 (2.72 - 4.93) | 3.55 (2.64 – 4.78) |
| 28-<33 weeks | 132 (2.7%) | 2.48 (2.08 – 2.96) | 2.15 (1.78 - 2.59) | 2.09 (1.73 – 2.52) |
| 33-<37 weeks | 337 (6.9%) | 1.33 (1.18 – 1.50) | 1.24 (1.09 - 1.41) | 1.22 (1.08 – 1.39) |
| 37-43 weeks | 4121 (84.4%) | 1.00 (ref) | 1.00 (ref) | 1.00 (ref) |
| **Parity (0.4% missing data)** | | | | |
| 0 | 2019 (41.4%) | 1.00 (ref) | 1.00 (ref) | 1.00 (ref) |
| ≥1 | 2845 (58.3%) | 1.05 (0.99 – 1.11) | 1.10 (1.03 - 1.17) | 1.08 (1.01 – 1.15) |
| **Multiple births** | | | | |
| No | 4702 (96.3%) | 1.00 (ref) | 1.00 (ref) |  |
| Yes | 180 (3.7%) | 1.27 (1.09 – 1.48) | 0.98 (0.83 - 1.15) | 0.98 (0.83 - 1.16) |
| **Small for gestational age (<10th centile) (5.6% missing data)** | | | | |
| No | 3993 (81.8%) | 1.00 (ref) | 1.00 (ref) | 1.00 (ref) |
| Yes | 615 (12.6%) | 1.50 (1.37 – 1.64) | 1.39 (1.27 - 1.53) | 1.38 (1.25 – 1.51) |
| **Congenital anomalies** | | | | |
| None | 4366 (89.4%) | 1.00 (ref) | 1.00 (ref) | 1.00 (ref) |
| Minor | 60 (1.2%) | 1.86 (1.42 – 2.42) | 1.55 (1.19 - 2.03) | 1.54 (1.18 – 2.02) |
| Major | 456 (9.3%) | 2.78 (2.52 – 3.07) | 2.49 (2.26 - 2.76) | 2.48 (2.25 – 2.75) |
| **Maternal cigarette smoking at booking in for birth (67% missing data)** | | | | |
| No | 1167 (23.9%) | 1.00 (ref) | 1.00 (ref) | 1.00 (ref) |
| Yes | 442 (9.1%) | 1.36 (1.14 – 1.61) | 1.16 (0.96 - 1.39) | 1.14 (0.95 – 1.37 ) |
