# Supplementary material for: Adverse childhood experiences and child mental health: an electronic birth cohort study
Source: BMC Med. 2021 Aug 6;19:172. doi: 10.1186/s12916-021-02045-x (PMC8344166; doi:10.1186/s12916-021-02045-x)
Supplement: Supplementary file 8 — Additional file 8: Table 6. Three-way cross-tabulation of ACEs, Deprivation and Any Mental Health (excluding developmental delay). [file 12916_2021_2045_MOESM8_ESM.docx]

**Additional File 8: Table 6 - Three-way cross-tabulation of ACEs, Deprivation and Any Mental Health (excluding developmental delay)**

|  | | **Any mental health (excluding developmental delay)** | |
| --- | --- | --- | --- |
|  | **ACE - Alcohol hospital admission or GP diagnosis** | **No** | **Yes** |
| **(1) Least Deprived** | No | 30,354 (98.6%) | 439 (1.4%) |
|  | Yes | 2856 (98.1%) | 55 (1.9%) |
| **(2) Deprived** | No | 30105 (98.4%) | 499 (1.6%) |
|  | Yes | 3976 (97.8%) | 91 (2.2%) |
| **(3) Deprived** | No | 31333 (98.3%) | 550 (1.7%) |
|  | Yes | 5237 (97.7%) | 125 (2.3%) |
| **(4) Deprived** | No | 31973 (98.2%) | 593 (1.8%) |
|  | Yes | 5237 (97.7%) | 125 (2.3%) |
| **(5) Most Deprived** | No | 34808 (97.9%) | 757 (2.1%) |
|  | Yes | 9760 (97.3%) | 270 (2.7%) |
|  | **ACE – Common Mental Health Disorder** | **No** | **Yes** |
| **(1) Least Deprived** | No | 19157 (99.0%) | 198 (1.0%) |
|  | Yes | 14053 (97.9%) | 296 (2.1%) |
| **(2) Deprived** | No | 18811 (98.7%) | 245 (1.3%) |
|  | Yes | 15270 (97.8%) | 345 (2.2%) |
| **(3) Deprived** | No | 18760 (98.8%) | 233 (1.2%) |
|  | Yes | 17810 (97.6%) | 442 (2.4%) |
| **(4) Deprived** | No | 18598 (98.7%) | 240 (1.3%) |
|  | Yes | 20102 (97.4%) | 532 (2.6%) |
| **(5) Most Deprived** | No | 19581 (98.5%) | 311 (1.5%) |
|  | Yes | 24717 (97.2%) | 716 (2.8%) |
|  | **ACE – Serious Mental Illness** | **No** | **Yes** |
| **(1) Least Deprived** | No | 32908 (98.5%) | 490 (1.5%) |
|  | Yes | ~306 (~100%) | <5 |
| **(2) Deprived** | No | 33675 (98.3%) | 580 (1.7%) |
|  | Yes | 406 (97.6%) | 10 (2.4%) |
| **(3) Deprived** | No | 36073 (98.2%) | 666 (1.8%) |
|  | Yes | 497 (98.2%) | 9 (1.8%) |
| **(4) Deprived** | No | 38027 (98.1%) | 740 (1.9%) |
|  | Yes | 673 (95.5%) | 32 (4.5%) |
| **(5) Most Deprived** | No | 42626 (97.8%) | 989 (2.2%) |
|  | Yes | 942 (96.1%) | 38 (3.9%) |
|  | **ACE – Victimisation** | **No** | **Yes** |
| **(1) Least Deprived** | No | 33026 (98.5%) | 486 (1.5%) |
|  | Yes | 184 (95.8%) | 8 (4.2%) |
| **(2) Deprived** | No | 33774 (98.3%) | 580 (1.7%) |
|  | Yes | 307 (96.3%) | 10 (3.2%) |
| **(3) Deprived** | No | 36243 (98.2%) | 658 (1.8%) |
|  | Yes | 327 (95.1%) | 17 (4.9%) |
| **(4) Deprived** | No | 38302 (98.1%) | 753 (1.9%) |
|  | Yes | 398 (95.4%) | 19 (4.6%) |
| **(5) Most Deprived** | No | 43790 (97.8%) | 981 (2.2%) |
|  | Yes | 778 (94.4%) | 46 (5.6%) |
|  | **ACE – Death** | **No** | **Yes** |
| **(1) Least Deprived** | No | 32128 (98.6%) | 469 (1.4%) |
|  | Yes | 1082 (97.7%) | 25 (2.3%) |
| **(2) Deprived** | No | 32692 (98.3%) | 558 (1.7%) |
|  | Yes | 1389 (97.7%) | 32 (2.3%) |
| **(3) Deprived** | No | 35101 (98.2%) | 631 (1.8%) |
|  | Yes | 1469 (97.1%) | 44 (2.9%) |
| **(4) Deprived** | No | 37021 (98.1%) | 723 (1.9%) |
|  | Yes | 1679 (97.2%) | 49 (2.8%) |
| **(5) Most Deprived** | No | 42537 (97.8%) | 972 (2.2%) |
|  | Yes | 2031 (97.4%) | 55 (2.6%) |
