# Supplementary material for: Adverse childhood experiences and child mental health: an electronic birth cohort study
Source: BMC Med. 2021 Aug 6;19:172. doi: 10.1186/s12916-021-02045-x (PMC8344166; doi:10.1186/s12916-021-02045-x)
Supplement: Supplementary file 9 — Additional file 9: Table 7. Three-way cross-tabulation of ACEs, Deprivation and Developmental Delay. [file 12916_2021_2045_MOESM9_ESM.docx]

**Additional File 9: Table 7 - Three-way cross-tabulation of ACEs, Deprivation and Developmental Delay**

|  | | **Developmental Delay** | |
| --- | --- | --- | --- |
|  | **ACE - Alcohol hospital admission or GP diagnosis** | **No** | **Yes** |
| **(1) Least Deprived** | No | 30186 (98.0%) | 607 (2.0%) |
|  | Yes | 2831 (97.3%) | 80 (2.7%) |
| **(2) Deprived** | No | 29933 (97.8%) | 671 (2.2%) |
|  | Yes | 3948 (97.1%) | 119 (2.9%) |
| **(3) Deprived** | No | 31115 (97.6%) | 768 (2.4%) |
|  | Yes | 5176 (96.5%) | 186 (3.5%) |
| **(4) Deprived** | No | 31774 (97.6%) | 792 (2.4%) |
|  | Yes | 6678 (96.7%) | 792 (2.4%) |
| **(5) Most Deprived** | No | 34521 (97.1%) | 1044 (2.9%) |
|  | Yes | 9656 (96.3%) | 374 (3.7%) |
|  | **ACE – Common Mental Health Disorder** | **No** | **Yes** |
| **(1) Least Deprived** | No | 19058 (98.5%) | 297 (1.5%) |
|  | Yes | 13959 (97.3%) | 390 (2.7%) |
| **(2) Deprived** | No | 18749 (98.4%) | 307 (1.6%) |
|  | Yes | 15132 (96.9%) | 483 (3.1%) |
| **(3) Deprived** | No | 18635 (98.1%) | 358 (1.9%) |
|  | Yes | 17656 (96.7%) | 596 (3.3%) |
| **(4) Deprived** | No | 18465 (98.0%) | 373 (2.0%) |
|  | Yes | 19987 (96.9%) | 647 (3.1%) |
| **(5) Most Deprived** | No | 19665 (97.5%) | 497 (2.5%) |
|  | Yes | 24512 (96.4%) | 921 (3.6%) |
|  | **ACE – Serious Mental Illness** | **No** | **Yes** |
| **(1) Least Deprived** | No | 32716 (98.0%) | 682 (2.0%) |
|  | Yes | 301 (98.4%) | 5 (1.6%) |
| **(2) Deprived** | No | 33476 (97.7%) | 779 (2.3%) |
|  | Yes | 405 (97.4%) | 11 (2.6%) |
| **(3) Deprived** | No | 35804 (97.5%) | 935 (2.5%) |
|  | Yes | 487 (96.2%) | 19 (3.8%) |
| **(4) Deprived** | No | 37775 (97.4%) | 992 (2.6%) |
|  | Yes | 677 (96.0%) | 28 (4.0%) |
| **(5) Most Deprived** | No | 43228 (96.9%) | 1387 (3.1%) |
|  | Yes | 949 (96.8%) | 31 (3.2%) |
|  | **ACE – Victimisation** | **No** | **Yes** |
| **(1) Least Deprived** | No | 32836 (98.0%) | 676 (2.0%) |
|  | Yes | 181 (94.3%) | 11 (5.7%) |
| **(2) Deprived** | No | 33578 (97.7%) | 776 (2.3%) |
|  | Yes | 303 (95.6%) | 14 (4.4%) |
| **(3) Deprived** | No | 35972 (97.5%) | 929 (2.5%) |
|  | Yes | 319 (92.7%) | 25 (7.3%) |
| **(4) Deprived** | No | 38066 (97.5%) | 989 (2.5%) |
|  | Yes | 319 (92.7%) | 25 (7.3%) |
| **(5) Most Deprived** | No | 43418 (97.0%) | 1353 (3.0%) |
|  | Yes | 759 (92.1%) | 65 (7.9%) |
|  | **ACE – Death** | **No** | **Yes** |
| **(1) Least Deprived** | No | 31934 (98.0%) | 663 (2.0%) |
|  | Yes | 1083 (97.8%) | 24 (2.2%) |
| **(2) Deprived** | No | 32499 (97.7%) | 751 (2.3%) |
|  | Yes | 1382 (97.3%) | 39 (2.7%) |
| **(3) Deprived** | No | 34829 (97.5%) | 903 (2.5%) |
|  | Yes | 1462 (96.6%) | 51 (3.4%) |
| **(4) Deprived** | No | 36785 (97.5%) | 959 (2.5%) |
|  | Yes | 1667 (96.5%) | 61 (3.5%) |
| **(5) Most Deprived** | No | 42185 (97.0%) | 1324 (3.0%) |
|  | Yes | 1992 (95.5%) | 94 (4.5%) |
