# Supplementary material for: Adverse childhood experiences and child mental health: an electronic birth cohort study
Source: BMC Med. 2021 Aug 6;19:172. doi: 10.1186/s12916-021-02045-x (PMC8344166; doi:10.1186/s12916-021-02045-x)
Supplement: Supplementary file 10 — Additional file 10: Table 8. Adult Mental Health codes for Common Mental Disorder (CMD) and Serious Mental Illness (SMI). [file 12916_2021_2045_MOESM10_ESM.docx]

**Additional File 10: Table 8 - Adult Mental Health codes for Common Mental Disorder (CMD) and Serious Mental Illness (SMI)**

| **Adult Common Mental Disorder Codes** | **Adult Serious Mental Illness Codes** |
| --- | --- |
| **SSRI**  event_cd like 'da3%' or  event_cd like 'da4%' or  event_cd like 'da5%' or  event_cd like 'da6%' or  event_cd like 'da9%' or  event_cd like 'daC%' or  **Tricyclics**  event_cd like 'd7%' or  (event_cd like 'd91%' and event_cd not in ('d915.')) or  **Others**  event_cd like 'da1%' OR  event_cd like 'da2%' OR  event_cd like 'da7%' OR  event_cd like 'daA%' OR  event_cd like 'daB%' OR  event_cd like 'daD%' or  **Duloxetine - gde%**  event_cd like 'gde%' or  **MOAIs**  event_cd like 'd8%' or  --parstelin  event_cd in ('d915.')  **Anxiolytics**  or (event_cd like 'd2%')  **Hypnotics**  or (event_cd like'd1%')) | E10% Schizophrenic disorders  E110% Manic disorder, single episode  E111% Recurrent manic episodes  E1124 Single major depressive episode, severe, with psychotic disorders  E1134 Recurrent major depressive episodes, severe, with psychotic disorders  E114% Bipolar affective disorder, currently manic  E115% Bipolar affective disorder, currently depressed  E116% Mixed bipolar affective disorder  E117% Unspecified bipolar affective disorder  E11y. Other and unspecified manic-depressive psychoses  E11y0 Unspecified manic-depressive psychoses  E11y1 Atypical manic disorder  E11y3 Other mixed manic-depressive psychoses  E11yz Other and unspecified manic-depressive psychoses NOS  E11z. Other and unspecified affective psychoses  E11z0 Unspecified affective psychoses NOS  E11zz Other affective psychotic disorders NOS  E12% Paranoid states  E13.. Other nonorganic psychoses  E130. Reactive depressive psychotic disorders  E131. Acute hysterical psychotic disorders  E132. Reactive confusion  E133. Acute paranoid reaction  E134. Psychogenic paranoid psychotic disorders  E13y. Other reactive psychoses  E13y0 Psychogenic stupor  E13y1 Brief reactive psychotic disorders  E13yz Other reactive psychoses NOS  E13z. Nonorganic psychotic disorders NOS  E2122 Schizotypal personality  Eu2% [X]Schizophrenia, schizotypal and delusional disorders  Eu30% [X]Manic episode  Eu31% [X]Bipolar affective disorder  Eu323 Severe depressive episode with psychotic symptoms  Eu333 [X] Recurrent depressive disorder, current episode severe with psychotic symptoms |
