# Supplementary material for: Adverse childhood experiences and child mental health: an electronic birth cohort study
Source: BMC Med. 2021 Aug 6;19:172. doi: 10.1186/s12916-021-02045-x (PMC8344166; doi:10.1186/s12916-021-02045-x)
Supplement: Supplementary file 11 — Additional file 11: Table 9. Codes for Externalising and Internalising categories. [file 12916_2021_2045_MOESM11_ESM.docx]

**Additional File 11: Table 9 - Codes for Externalising and Internalising categories**

| **Codes for Externalising category** | | **Codes for Internalising category** | |
| --- | --- | --- | --- |
| E2C.. | Disturbance of conduct NEC | E2781 | Tension headache |
| E2Czz | Disturbance of conduct NOS | E2Dz. | Childhood and adolescent emotion disorder NOS |
| E2Cz. | Unspecified disturbance of conduct | E205. | Neurasthenia - nervous debility |
| E2C0. | Aggressive unsocial conduct disorder | E200. | Anxiety states |
| E2C1. | Nonaggressive unsocial conduct disorder | E2001 | Panic disorder |
| E2C10 | Unsocial childhood truancy | E2748 | Night terrors |
| Eu91z | [X] Conduct disorder, unspecified | E2741 | Transient insomnia |
| Eu91. | [X] Conduct disorders | E2C01 | Anger reaction |
| Eu912 | [X] Socialized conduct disorder | E2003 | Anxiety with depression |
| E2C0z | Aggressive unsocial conduct disorder NOS | E2749 | Nightmares |
|  |  | E274. | Non-organic sleep disorders |
|  |  | E2900 | Grief reaction |
|  |  | E276. | Non-organic enuresis |
|  |  | E200z | Anxiety state NOS |
|  |  | E2D.. | Disturbance of emotion specific to childhood and adolescence |
|  |  | E203. | Obsessive-compulsive disorders |
|  |  | E28.. | Acute reaction to stress |
|  |  | E2000 | Anxiety state unspecified |
|  |  | E202. | Phobic disorders |
|  |  | E2C00 | Aggressive outburst |
|  |  | E202A | Fear of flying |
|  |  | E2D2z | Childhood and adolescent sensitivity disturbance NOS |
|  |  | E2742 | Persistent insomnia |
|  |  | E2760 | Non-organic primary enuresis |
|  |  | E2645 | Psychogenic constipation |
|  |  | E2757 | Psychogenic polydipsia |
|  |  | E274D | Repetitive intrusions of sleep |
|  |  | E2D0. | Disturbance of anxiety and fearfulness childhood/adolescent |
|  |  | E2141 | Obsessional personality |
|  |  | E2005 | Recurrent anxiety |
|  |  | E207. | Hypochondriasis |
|  |  | E29.. | Adjustment reaction |
|  |  | E2920 | Separation anxiety disorder |
|  |  | E2002 | Generalised anxiety disorder |
|  |  | E2020 | Phobia unspecified |
|  |  | E2756 | Non-organic loss of appetite |
|  |  | E2761 | Non-organic secondary enuresis |
|  |  | E2D1. | Childhood and adolescence disturbance of unhappiness |
|  |  | E2021 | Agoraphobia with panic attacks |
|  |  | E2613 | Psychogenic hyperventilation |
|  |  | E2721 | Transient childhood tic |
|  |  | E2027 | Animal phobia |
|  |  | E2642 | Cyclical vomiting - psychogenic |
|  |  | E2643 | Psychogenic diarrhoea |
|  |  | E2720 | Tic disorder unspecified |
|  |  | E277. | Non-organic encopresis |
|  |  | E28z. | Acute stress reaction NOS |
|  |  | E274E | Short-sleeper |
|  |  | E272z | Tic NOS |
|  |  | E203z | Obsessive-compulsive disorder NOS |
|  |  | E2015 | Hysterical seizures |
|  |  | E202C | Dental phobia |
|  |  | E202z | Phobic disorder NOS |
|  |  | E2930 | Adjustment reaction with aggression |
|  |  | E2D20 | Childhood and adolescent disturbance with shyness |
|  |  | E2004 | Chronic anxiety |
|  |  | E2611 | Psychogenic cough |
|  |  | E2Dy. | Other childhood and adolescent emotional problems |
|  |  | E2740 | Unspecified non-organic sleep disorder |
|  |  | E2031 | Obsessional neurosis |
|  |  | E26z. | Psychosomatic disorder NOS |
|  |  | E274y | Other non-organic sleep disorder |
|  |  | E274z | Non-organic sleep disorder NOS |
|  |  | E2D0z | Disturbance anxiety and fearfulness childhood/adolescent NOS |
|  |  | E2754 | Psychogenic vomiting NOS |
|  |  | E2D3. | Childhood and adolescent relationship problem |
|  |  | E2640 | Psychogenic aerophagy |
|  |  | E280. | Acute panic state due to acute stress reaction |
|  |  | E2831 | Acute posttrauma stress state |
|  |  | Eu42. | [X] Obsessive - compulsive disorder |
|  |  | Eu431 | [X] Post - traumatic stress disorder |
|  |  | Eu41z | [X] Anxiety disorder, unspecified |
|  |  | Eu40z | [X] Phobic anxiety disorder, unspecified |
|  |  | Eu410 | [X] Panic disorder [episodic paroxysmal anxiety] |
|  |  | Eu403 | [X] Needle phobia |
|  |  | Eu411 | [X] Generalized anxiety disorder |
|  |  | Eu513 | [X] Sleepwalking |
|  |  | Eu633 | [X] Trichotillomania |
|  |  | Eu402 | [X] Specific (isolated) phobias |
|  |  | Eu95. | [X] Tic disorders |
|  |  | Eu401 | [X] Social phobias |
|  |  | Eu41. | [X] Other anxiety disorders |
|  |  | Eu51z | [X] Nonorganic sleep disorder, unspecified |
|  |  | Eu412 | [X] Mixed anxiety and depressive disorder |
|  |  | Eu515 | [X] Nightmares |
|  |  | Eu40. | [X] Phobic anxiety disorders |
|  |  | Eu430 | [X] Acute stress reaction |
|  |  | Eu932 | [X] Social anxiety disorder of childhood |
|  |  | Eu432 | [X] Adjustment disorders |
|  |  | Eu421 | [X] Predominantly compulsive acts [obsessional rituals] |
|  |  | Eu45y | [X] Other somatoform disorders |
|  |  | Eu930 | [X] Separation anxiety disorder of childhood |
|  |  | Eu452 | [X] Hypochondriacal disorder |
|  |  | Eu505 | [X] Vomiting associated with other psychological disturbances |
|  |  | Eu94z | [X] Childhood disorder of social functioning, unspecified |
|  |  | Eu951 | [X] Chronic motor or vocal tic disorder |
|  |  | Eu514 | [X] Sleep terrors |
|  |  | Eu9y0 | [X] Nonorganic enuresis |
|  |  | Eu9y1 | [X] Nonorganic encopresis |
|  |  | E2B.. | Depressive disorder NEC |
|  |  | E204. | Neurotic depression reactive type |
|  |  | E2B1. | Chronic depression |
|  |  | E1137 | Recurrent depression |
|  |  | Eu32z | [X] Depressive episode, unspecified |
|  |  | Eu32. | [X] Depressive episode |
|  |  | Eu321 | [X] Moderate depressive episode |
|  |  | Eu320 | [X] Mild depressive episode |
|  |  | Eu324 | [X] Mild depression |
|  |  | Eu33. | [X] Recurrent depressive disorder |
