# Supplementary material for: Adverse childhood experiences and child mental health: an electronic birth cohort study
Source: BMC Med. 2021 Aug 6;19:172. doi: 10.1186/s12916-021-02045-x (PMC8344166; doi:10.1186/s12916-021-02045-x)
Supplement: Supplementary file 12 — Additional file 12: Table 10. Codes for Other and Developmental delay. [file 12916_2021_2045_MOESM12_ESM.docx]

**Additional File 12: Table 10 - Codes for Other and Developmental delay**

| **Codes for Other category** | | **Codes for Developmental delay category** | |
| --- | --- | --- | --- |
| E275. | Other and unspecified non-organic eating disorders | E2F3. | Speech or language developmental disorder |
| E271. | Anorexia nervosa | E2F.. | Specific delays in development |
| E2751 | Bulimia (non-organic overeating) | E2E0. | Child attention deficit disorder |
| E2750 | Unspecified non-organic eating disorder | E2Fy. | Other development delays |
| E275z | Non-organic eating disorder NOS | E2C12 | Tantrums |
| Eu50. | [X] Eating disorders | E140. | Infantile autism |
| Eu50z | [X] Eating disorder, unspecified | E2Cy0 | Breath holder |
| Eu502 | [X] Bulimia nervosa | E2F4. | Coordination disorder (dyspraxia) |
| Eu50y | [X] Other eating disorders | E2F5. | Mixed development disorder |
| Eu500 | [X] Anorexia nervosa | E2E01 | Attention deficit with hyperactivity |
| E112. | Single major depressive episode | E2Fz. | Developmental disorder NOS |
| E213. | Explosive personality disorder | E2E.. | Childhood hyperkinetic syndrome |
| E13z. | Nonorganic psychosis NOS | E2F3z | Speech or language developmental disorder NOS |
| E2016 | Other conversion disorder | E270. | Stammering or stuttering |
| E21.. | Personality disorders | E2723 | Gilles de la Tourette's disorder |
| E113. | Recurrent major depressive episode | E272. | Tics |
| E21yz | Other personality disorder NOS | E27z2 | Lisping |
| E103. | Paranoid schizophrenia | E2731 | Head-banging |
| E135. | Agitated depression | E2F30 | Developmental aphasia |
| E2028 | Claustrophobia | E2747 | Somnambulism - sleep walking |
| E2151 | Munchausen's syndrome | E27z4 | Nail-biting |
| E273. | Stereotyped repetitive movements | E2F2. | Other specific learning difficulty |
| Eu90. | [X] Hyperkinetic disorders | E2F02 | Developmental dyslexia |
| Eu322 | [X] Severe depressive episode without psychotic symptoms | E2752 | Pica |
| Eu400 | [X] Agoraphobia | E27z5 | Thumb-sucking |
| Eu445 | [X] Dissociative convulsions | E3... | Mental retardation |
| Eu2z. | [X] Unspecified nonorganic psychosis | E2E0z | Child attention deficit disorder NOS |
| Eu04. | [X] Delirium, not induced by alcohol+other psychoactive subs | E2E2. | Hyperkinetic conduct disorder |
| Eu31. | [X] Bipolar affective disorder | E2E00 | Attention deficit without hyperactivity |
| Eu220 | [X] Delusional disorder | E2Dy0 | Childhood and adolescent oppositional disorder |
| Eu3.. | [X] Mood - affective disorders | E2C2. | Socialised conduct disorder |
| Eu323 | [X] Severe depressive episode with psychotic symptoms | E30.. | Mild mental retardation, IQ in range 50-70 |
| Eu341 | [X] Dysthymia | E2F00 | Reading disorder unspecified |
| Eu422 | [X] Mixed obsessional thoughts and acts | E140z | Infantile autism NOS |
| Eu44. | [X] Dissociative [conversion] disorders | E2722 | Chronic motor tic disorder |
| Eu90z | [X] Hyperkinetic disorder, unspecified | E2Ez. | Hyperkinetic syndrome NOS |
| E.... | Mental disorders | E2F0. | Specific reading disorder |
| E2A2. | Post-concussion syndrome | E3z.. | Mental retardation NOS |
| E2755 | Non-organic infant feeding disturbance | E1400 | Active infantile autism |
| E2273 | Impotence | E2023 | Social phobia, fear of eating in public |
| E030. | Acute confusional state | E2D22 | Childhood and adolescent disturbance with elective mutism |
| E26y0 | Bruxism (teeth grinding) | E2F1. | Dyscalculia |
| E27z3 | Masturbation | Eu900 | [X] Disturbance of activity and attention |
| E27z0 | Hair plucking | Eu845 | [X] Asperger's syndrome |
| E2C11 | Solitary stealing | Eu840 | [X] Childhood autism |
| Eu... | [X] Mental and behavioural disorders | Eu81z | [X] Developmental disorder of scholastic skills, unspecified |
| Eu453 | [X] Somatoform autonomic dysfunction | Eu84z | [X] Pervasive developmental disorder, unspecified |
| Eu9y2 | [X] Feeding disorder of infancy and childhood | Eu9y7 | [X] Attention deficit disorder |
| Eu9y3 | [X] Pica of infancy and childhood | Eu82. | [X] Specific developmental disorder of motor function |
| Eu530 | [X] Mild mental/behav disorder assoc with the puerperium NEC | Eu913 | [X] Oppositional defiant disorder |
| Eu842 | [X] Rett's syndrome | Eu80. | [X] Specific developmental disorders of speech and language |
| Eu85. | Global developmental delay | Eu9.. | [X] Behavioural/emotional disorders onset childhood/adolescence |
| Eu814 | Moderate learning disability | Eu953 | [X] Involuntary excessive blinking |
| Eu816 | Mild learning disability | Eu801 | [X] Expressive language disorder |
| Eu815 | Severe learning disability | Eu80z | [X] Developmental disorder of speech and language unspecified |
|  |  | Eu940 | [X] Elective mutism |
|  |  | Eu841 | [X] Atypical autism |
|  |  | Eu84. | [X] Pervasive developmental disorders |
|  |  | Eu800 | [X] Specific speech articulation disorder |
|  |  | Eu802 | [X] Receptive language disorder |
|  |  | Eu902 | [X] Deficits in attention, motor control and perception |
|  |  | Eu941 | [X] Reactive attachment disorder of childhood |
|  |  | Eu63. | [X] Habit and impulse disorders |
|  |  | Eu80y | [X] Other developmental disorders of speech and language |
|  |  | Eu81. | [X] Specific developmental disorders of scholastic skills |
|  |  | Eu9y5 | [X] Stuttering [stammering] |
|  |  | Eu810 | [X] Specific reading disorder |
|  |  | Eu92. | [X] Mixed disorders of conduct and emotions |
|  |  | Eu901 | [X] Hyperkinetic conduct disorder |
|  |  | Eu83. | [X] Mixed specific developmental disorders |
