# Supplementary material for: Adverse childhood experiences and child mental health: an electronic birth cohort study
Source: BMC Med. 2021 Aug 6;19:172. doi: 10.1186/s12916-021-02045-x (PMC8344166; doi:10.1186/s12916-021-02045-x)
Supplement: Supplementary file 13 — Additional file 13: Table 11. ICD-10 codes for victimisation, taken from Lee, Gonzalez-Izquierod, and Gilbert (2012) [27]. [file 12916_2021_2045_MOESM13_ESM.docx]

**Additional File 13:** **Table 11 - ICD-10 codes for victimisation, taken from Lee, Gonzalez-Izquierod, and Gilbert (2012)**

| **Description** | **ICD-10 code** |
| --- | --- |
| **1. Maltreatment** | |
| Maltreatment syndromes | T74 |
| Perpetrator of neglect and other maltreatment syndromes | Y06, Y07 |
| **2. Assault** | |
| Assault by bodily force | Y04, Y05 |
| Other types of assault | X85 - Y03, Y08 - Y09 |
| **3. Undetermined cause** | |
| Events of undetermined intent | Y10 - Y34 |
| Blood alcohol and blood-drug tests | Z04.0 |
| Examination and observation following other inflicted injury | Z04.5 |
| Examination and observation for other reasons: request for expert evidence | Z04.8 |
| **4. Adverse social circumstances** | |
| Problems related to social environment | Z60 |
| Problems related to negative life events in childhood | Z61 |
| Other problems related to upbringing | Z62 |
| Other problems related to primary support group | Z63 |
| Problems related to other legal circumstances | Z65.3 |
| Problems related to lifestyle (Except Z72.0 Tobacco use) | Z72 |
| Problems related to care-provider dependency | Z74 |
| Health supervision and care of foundling | Z76.1 |
| Health supervision and care of other healthy infant and child | Z76.2 |
| Family history of mental and behavioural disorders | Z81 |
| Personal history of other mental and behavioural disorders | Z86.5 |
| Personal history of other physical trauma | Z91.6 |
| Personal history of other specified risk-factors, not elsewhere classified | Z91.8 |
