# Supplementary material for: Adverse childhood experiences and child mental health: an electronic birth cohort study
Source: BMC Med. 2021 Aug 6;19:172. doi: 10.1186/s12916-021-02045-x (PMC8344166; doi:10.1186/s12916-021-02045-x)
Supplement: Supplementary file 14 — Additional file 14: Table 12. Table of national population statistics. [file 12916_2021_2045_MOESM14_ESM.docx]

**Additional File 14: Table 12 - Table of national population statistics**

**^a^Deprivation and health – report for the National Public Health Service for Wales 2004;**

**^b^Welsh data from the UK Census 2001 at** [**https://statswales.gov.wales/**](https://statswales.gov.wales/) **;**

**^c^Births in Wales 2001 - 2011: Data from the National Community Child Health Database 2012.**

|  | **Total** | |
| --- | --- | --- |
|  | **n (%)** | |
| Townsend Deprivation quintile: from 2003, child’s age 0 – 14 years old^a^ |  |  |
| 1 - least deprived | - | (19.3) |
| 2 | - | (19.3) |
| 3 | - | (19.3) |
| 4 | - | (20.5) |
| 5 - most deprived | - | (21.7) |
| Sex: from 2001, child’s age 0 – 14 years old^b^ |  |  |
| Male | 281767 | (51.3) |
| Female | 267437 | (48.7) |
| Breastfeeding at birth: Welsh residents 2011^c^ |  |  |
| No | 14469 | (40.5) |
| Yes | 18062 | (50.6) |
| no answer | 3151 | (8.8) |
| Maternal age at childbirth: Welsh residents 2011 ^c^ |  |  |
| <16 | 57 | (0.2) |
| 16-19 | 2409 | (6.8) |
| 19-24 | 8115 | (22.7) |
| 25-29 years | 10268 | (28.8) |
| 30-34 | 9107 | (25.5) |
| 35+ | 5722 | (16.0) |
| no answer | 4 | (0.01) |
| Gestational age at birth: Welsh residents 2011 ^c^ |  |  |
| 20-<32 weeks | 443 | (1.2) |
| 32-<37 weeks | 2094 | (5.9) |
| 37-43 weeks | 32985 | (92.4) |
| no answer | 160 | (0.4) |
| Birthweight: Welsh residents 2011 ^c^ |  |  |
| Low: < 2500g | 2403 | (6.7) |
| Normal: ≥ 2500 - < 4000g | 28991 | (81.2) |
| High: ≥ 4000g | 4249 | (11.9) |
| no answer | 39 | (0.1) |
