# Supplementary material for: Adverse childhood experiences and child mental health: an electronic birth cohort study
Source: BMC Med. 2021 Aug 6;19:172. doi: 10.1186/s12916-021-02045-x (PMC8344166; doi:10.1186/s12916-021-02045-x)
Supplement: Supplementary file 15 — Additional file 15: Table 13. Moderation analysis for ACEs, deprivation, and child mental health. [file 12916_2021_2045_MOESM15_ESM.docx]

|  |  | **Any mental health** | | | **Developmental delay** | | |
| --- | --- | --- | --- | --- | --- | --- | --- |
|  |  | **cHR** | **Lower CI** | **Upper CI** | **cHR** | **Lower CI** | **Upper CI** |
| **Alcohol** | **2^nd^** | 1.03 | 0.64 | 1.64 | 1.33 | 0.88 | 2.00 |
|  | **3^rd^** | 0.92 | 0.59 | 1.44 | 1.03 | 0.69 | 1.54 |
|  | **4^th^** | 0.89 | 0.58 | 1.37 | 0.93 | 0.63 | 1.37 |
|  | **Most deprived** | 0.92 | 0.61 | 1.39 | 1.00 | 0.69 | 1.44 |
|  | | | | | | | |
| **CMD** | **2^nd^** | 1.01 | 0.78 | 1.30 | 1.00 | 0.81 | 1.25 |
|  | **3^rd^** | 1.19 | 0.93 | 1.53 | 0.94 | 0.77 | 1.16 |
|  | **4^th^** | 1.22 | 0.95 | 1.55 | 0.85 | 0.69 | 1.04 |
|  | **Most deprived** | 1.16 | 0.92 | 1.46 | 0.81 | 0.67 | 0.98 |
|  | | | | | | | |
| **SMI** | **2^nd^** | 1.62 | 0.33 | 8.07 | 2.74 | 0.30 | 24.86 |
|  | **3^rd^** | 0.44 | 0.06 | 3.12 | 2.97 | 0.36 | 24.22 |
|  | **4^th^** | 1.78 | 0.40 | 8.01 | 2.92 | 0.37 | 23.23 |
|  | **Most deprived** | 2.10 | 0.49 | 9.04 | 2.57 | 0.33 | 20.08 |
|  | | | | | | | |
| **Death** | **2^nd^** | 0.85 | 0.23 | 3.18 | 0.75 | 0.24 | 2.34 |
|  | **3^rd^** | 1.37 | 0.42 | 4.49 | 0.70 | 0.23 | 2.08 |
|  | **4^th^** | 0.85 | 0.25 | 2.93 | 0.72 | 0.25 | 2.10 |
|  | **Most deprived** | 1.34 | 0.44 | 4.09 | 0.80 | 0.30 | 2.15 |
|  | | | | | | | |
| **Victimisation** | **2^nd^** | 0.25 | 0.06 | 1.04 | 1.22 | 0.24 | 6.32 |
|  | **3^rd^** | 0.36 | 0.10 | 1.25 | 2.64 | 0.59 | 11.83 |
|  | **4^th^** | 0.32 | 0.10 | 1.05 | 1.54 | 0.33 | 7.17 |
|  | **Most deprived** | 0.38 | 0.14 | 1.08 | 1.61 | 0.37 | 6.95 |

**Additional File 15: Table 13 - Moderation analysis for ACEs, deprivation, and child mental health**
